# Myofibrillogenesis Regulator‐1 Regulates the Ubiquitin Lysosomal Pathway of Notch3 Intracellular Domain Through E3 Ubiquitin‐Protein Ligase Itchy Homolog in the Metastasis of Non‐Small Cell Lung Cancer

**DOI:** 10.1002/advs.202306472

**Published:** 2024-02-11

**Authors:** Wenxia Zhao, Yang Li, Hanzeng Cheng, Mengyan Wang, Zhishuo Zhang, Meilian Cai, Cong Zhao, Xiaoming Xi, Xiaojun Zhao, Wuli Zhao, Yajun Yang, Rongguang Shao

**Affiliations:** ^1^ NHC Key Laboratory of Antibiotic Bioengineering, Laboratory of Oncology Institute of Medicinal Biotechnology Chinese Academy of Medical Sciences & Peking Union Medical College Beijing 100050 Beijing P. R. China; ^2^ Beijing Key Laboratory of Active Substance Discovery and Druggability Evaluation, Institute of Materia Medica Peking Union Medical College and Chinese Academy of Medical Sciences Beijing 100050 P. R. China; ^3^ Zhujiang Hospital Southern Medical University Guangzhou Guangdong 510280 P. R. China; ^4^ Department of Emergency Xinhua Hospital Shanghai Jiaotong University School of Medicine Shanghai 200092 P. R. China; ^5^ Department of Organ Transplantation and Hepatobiliary Surgery The First Hospital of China Medical University Shenyang Liaoning 110001 P. R. China

**Keywords:** myofibrillogenesis regulator‐1, non‐small cell lung cancer, notch signaling pathway, notch3, NICD3 degradation, peptide antagonist, protein stability

## Abstract

Myofibrillogenesis regulator‐1 (MR‐1) is a multifunctional protein involved in the development of various human tumors. The study is the first to report the promoting effect of MR‐1 on the development and metastasis of non‐small cell lung cancer (NSCLC). MR‐1 is upregulated in NSCLC and positively associated with poor prognosis. The overexpression of MR‐1 promotes the metastasis of NSCLC cells by stabilizing the expression of Notch3‐ICD (NICD3) in the cytoplasm through enrichment analysis, in vitro and in vivo experimental researches. And Notch3 signaling can upregulate many genes related to metastasis. The stabilizing effect of MR‐1 on NICD3 is achieved through the mono‐ubiquitin lysosomal pathway and the specific E3 ubiquitin ligase is Itchy homolog (ITCH). There is a certain interaction between MR‐1 and NICD3. Elevated MR‐1 can affect the level of ITCH phosphorylation, reduce its E3 enzyme activity, and thus lead to reduce the ubiquitination and degradation of NICD3. Interference with the interaction between MR‐1 and NICD3 can increase the degradation of NICD3 and impair the metastatic ability of NSCLC cells, which is a previously overlooked treatment option in NSCLC. In summary, interference with the interaction between MR‐1 and NICD3 in the progression of lung cancer may be a promising therapeutic target.

## Graphical abstract

The novel cancer related multifunctional protein MR‐1 (Myofrillogenes regulator‐1) can stabilize the expression of Notch3‐ICD (NICD3) in the cytoplasm through ITCH, which can upregulate many genes related to metastasis to promote the metastasis of NSCLC cells. Interference with the interaction between MR‐1 and NICD3 can increase the degradation of NICD3 and impair the metastatic ability of NSCLC cells.

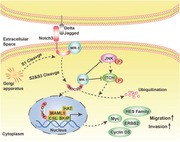

## 1 Introduction

Lung cancer is the type of cancer with the highest mortality rate in humans. According to cancer data statistics from the United States and China, the mortality rate of lung cancer has ranked first among both males and females in recent years. And non‐small cell lung cancer (NSCLC) is the main type of cancer in lung cancer, accounting for ≈85–90% of all cases.^[^ ^1^, ^2^, ^3^, ^4^ ^]^ For radical surgical treatment of lung cancer, only a small number of patients diagnosed with early lung tumors can accept. Moreover, the risk of postoperative recurrence is high.^[^ ^5^, ^6^ ^]^ The poor prognosis and clinical treatment difficulties of NSCLC are mainly attributed to the characteristics of metastasis obtained from NSCLC.^[^ ^7^ ^]^ The inevitable malignant development and late metastasis are unresolved clinical issues.

Human myofibrillogenesis regulator‐1 (MR‐1) is a novel functional protein also known as paroxysmal nonkinesigenic dyskinesia (*PNKD*) involved in the occurrence and progression of many diseases.^[^ ^8^ ^]^ Compared to other splicing forms, only MR‐1S (the short spliceosome of MR‐1, replaced by MR‐1 in the following description) was found to participate in cell function regulation as a tumor promoting protein, including high expression in ovarian cancer,^[^ ^9^, ^10^ ^]^ related to the poor prognosis of pancreatic cancer, liver cancer, and gastric cancer,^[^ ^11^, ^12^, ^13^ ^]^ promoting the adhesion and metastasis of liver cancer by activating Akt signaling pathway^[^ ^14^ ^]^ and promoting the proliferation of breast cancer by activating ERK signaling pathway.^[^ ^15^ ^]^ The above all suggest that MR‐1 has a promoting effect on various tumors. The Notch pathway is an evolutionarily conserved signaling pathway that involves various important cellular functions. The ECN (extracellular Notch domain) and NTM (Notch transmembrane fragment), formed by the first digestion of the single chain precursor molecule of Notch receptor protein by furin protease in Golgi apparatus, are bound together by a Ca^2+^ dependent non covalent bond to form a mature Notch receptor in the form of heterodimer and transported to the cell surface.^[^ ^16^ ^]^ The Notch ligand Delta on the cell surface binds to Notch receptors on adjacent cell membranes, initiating the signaling pathway. Notch receptor is digested for the second time under the action of tumor necrosis factor‐α‐convening enzyme (TACE) of ADAM (a disintegrin and metalloprotease) metalloproteinase family to release some extracellular fragments.^[^ ^17^ ^]^ Subsequently, through γ‐secretase, the third protease cleavage occurs, generating intracellular segments (NICDs) that are released from the membrane into the cytoplasm of the receptor cells.^[^ ^18^ ^]^ NICDs translocate into the nucleus, bind to CSL (CBF‐1, Suppressor of hairless, Lag), and recruit co activators including mastermind‐like family members (MAML) to regulate the expression of target genes.^[^ ^19^, ^20^, ^21^, ^22^, ^23^, ^24^ ^]^ Multiple studies have shown that dysfunction of the Notch signaling pathway is closely related to various lung diseases, including chronic obstructive pulmonary disease (COPD),^[^ ^25^ ^]^ asthma,^[^ ^26^ ^]^ idiopathic pulmonary fibrosis (IPF)^[^ ^27^, ^28^ ^]^ and pulmonary arterial hypertension (PAH).^[^ ^29^ ^]^ Especially Notch3 signaling has a high involvement in the development of acute and chronic lung diseases.^[^ ^30^ ^]^ In addition, more and more studies have shown that Notch3 has a certain promoting effect on the occurrence, development, and metastasis of lung cancer.^[^ ^31^, ^32^, ^33^, ^34^ ^]^ It has been reported that in pancreatic cancer, the nuclear expression of Notch3 and its target gene HEY‐1 can be used as its biomarker for diagnosis, prognosis and therapeutic effect.^[^ ^35^ ^]^ In non‐small cell lung cancer (NSCLC), canonical Wnt signaling activates Notch3, upregulating the expression of Notch3 protein and its target genes HES1 and HEYL mRNA level, thereby increasing cell proliferation and inhibiting apoptosis.^[^ ^32^ ^]^ Notch3 expression is also associated with the development of gastric cancer and intestinal/glandular differentiation of gastric cancer cells, indicating that it may be a favorable prognostic indicator.^[^ ^36^ ^]^

In existing research, the role and mechanism of MR‐1 in NSCLC are not yet clear. The purpose of this study is to explore the exact role and mechanism of MR‐1 in NSCLC. Some clinical studies use γ‐secretase inhibitors block Notch signaling in various human lung diseases.^[^ ^37^, ^38^ ^]^ This includes patients with refractory metastasis or locally advanced NSCLC, but these patients almost end up with treatment failure.^[^ ^39^, ^40^, ^41^ ^]^ It may be due to the lack of specificity of this treatment method for a single Notch receptor, leading to toxic side effects of treatment. So, we also purpose to search for better treatment strategies targeting Notch receptors.

In this study, we found that MR‐1 has an interaction with Notch3‐ICD (NICD3). MR‐1 can lead to high migration and invasion of NSCLC cells by inhibiting the ubiquitination and degradation of NICD3 mediated by the E3 ubiquitin ligase Itchy homolog (ITCH). Targeting NICD3 can also reduce cell metastasis ability by interfering with the interaction between MR‐1 and NICD3 with peptides. This study provides certain functional significance for the development of new targets and treatment methods for NSCLC treatment.

## 2 Results

### 2.1 The Upregulation of MR‐1 in NSCLC was Positively Correlated with Clinical Adverse Prognosis

**Figure 1:**
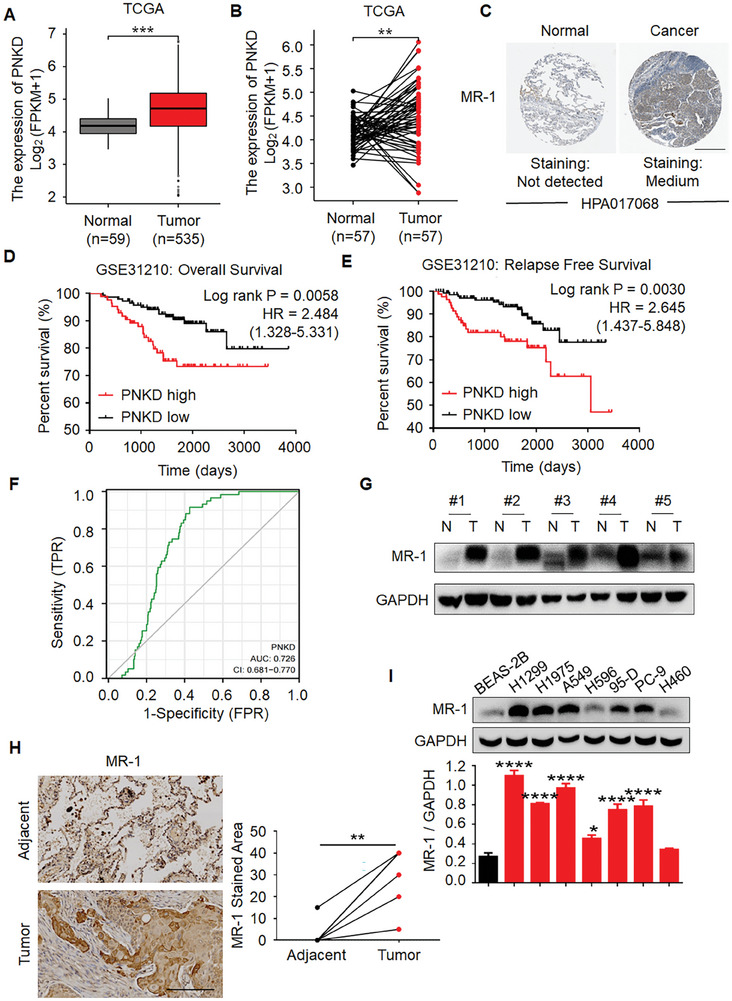
Upregulated MR‐1 in NSCLC correlates with poor prognosis. A,B) *PNKD* mRNA levels in tumor tissues and normal lung tissues from TCGA‐LUAD datasets. **p < 0.01; ***p < 0.001. C) MR‐1 protein levels in tumor tissues and normal lung tissues from HPA datasets. Scale bar, 400 µm. D,E) Overall survival (OS) and relapse free survival (RFS) curves analysis for 226 patients with NSCLC stratified by high and low *PNKD* expression in the GSE31210 data set. Log‐rank (Mantel‐Cox) test. **p < 0.01; ***p < 0.005. F) ROC curve of the diagnostic accuracy of *PNKD* in lung cancer from TCGA‐LUAD datasets. The horizontal axis represents the False Positive Rate (FPR), while the vertical axis represents the True Positive Rate (TPR). G) The expression of MR‐1 in NSCLC tissues and adjacent tissues of five groups of clinical samples. N, normal; T, tumor. H) IHC determined MR‐1 expression in NSCLC and adjacent tissues in TMA LUM961. Scale bar, 200 µm. Paired *t*‐test. **p < 0.01. I) Western blotting analysis of MR‐1 expression in seven NSCLC cell lines and the normal bronchial epithelial cells (BEAS‐2B). GAPDH was used as a loading control. One‐way ANOVA. The data are shown as mean ± SEM (*n* ≥ 3). *p < 0.05; ****p < 0.001.

To investigate the role of MR‐1 in NSCLC, we compared the most common subtype of NSCLC, lung adenocarcinoma (LUAD), in the TCGA database. The analysis of 594 paired and unpaired LUAD patients showed that the level of *PNKD* mRNA expression was higher in LUAD tissue compared to normal lung tissue (**Figure** **1**A, B). The Human Protein Atlas is an open access resource platform for human protein expression, which uses proteomics technology to identify differentially expressed tumor type specific protein expression patterns in specific types of tumors. Based on that the level of MR‐1 mRNA was highly expressed in lung cancer, we attempted to explore the protein expression pattern of MR‐1 in lung cancer by HPA. The results showed that MR‐1 is highly expressed in protein level of lung cancer compared to normal lung tissue (Figure 1C). To investigate the relationship between MR‐1 and clinical patient prognosis, we analyzed the expression profile of 226 NSCLC patients in the GEO database GSE31210 dataset and the survival analysis was performed in groups with *PNKD* as the median. The results showed that patients with high *PNKD* expression had poorer overall survival (OS) (HR = 2.484, 1.328‐5.331, Log rank *P* = 0.0058) and relapse free survival (RFS) (HR = 2.645, 1.437‐5.848, Log rank *P* = 0.0030) compared with those with low *PNKD* expression (Figure 1D, E). For OS, both groups had undefined median survival time. For RFS, the median survival time in the high expression group of PNKD was 3058 days, while the low expression group was undefined. Subsequently, we analyzed the TCGA database LUAD dataset through ROC curves and found that *PNKD* has certain diagnostic value for LUAD (AUC = 0.726, 95% CI = 0.681‐0.770) (Figure 1F), indicating that the high expression of *PNKD* has certain reference value for the differential diagnosis of LUAD. In addition, Western blot was performed on 6 NSCLC patients and their corresponding adjacent tissues collected from the First Affiliated Hospital of China Medical University, and the IHC tests were performed on 7 NSCLC patients (4 cases of adenosquamous carcinoma, 1 case of adenocarcinoma and 2 cases of squamous carcinoma) and their corresponding adjacent tissue arrays (Figure 1G, H) through TMA (LUM961). The results indicated that MR‐1 was highly expressed in tumor tissues compared with adjacent non‐tumor lung tissues. Consistent with the in vitro results of cell experiments, as shown in Figure 1I, the expression of MR‐1 was increased in NSCLC cells compared to human normal bronchial epithelial cells (BEAS‐2B). In summary, these results indicate that MR‐1 is highly expressed in NSCLC tissue and is detrimental to the prognosis of NSCLC patients.

### 2.2 MR‐1 Accelerated the Occurrence and Development of NSCLC in Transgenic Mice

In order to observe the role of high expression of MR‐1 in the occurrence and development of NSCLC disease more intuitively, we constructed a transgenic mouse model to accurately reflect the occurrence and development process of the disease.

*Cre*/*ERT2* mice are a kind of mice that contain fusion protein expression of Cre recombinase and estrogen receptor (ER) ligand binding region mutant. After tamoxifen induction, *Cre*/*ERT2* can enter the nucleus and activate Cre recombinase. This article used *sftpc‐Cre*/*ERT2* knockin mice (abbreviated as *c* transgenic mice). Carrying the IRES‐*cre* sequence in the 3′ ‐ UTR region of the *sftpc* gene (pulmonary surfactant protein C gene), Cre protein can be directionally expressed in type II alveolar epithelial cells in the process of tamoxifen induction. It can show efficient lung tissue specificity and is a commonly used tool mouse in the field of lung disease research. It can cause lung cancer (abbreviated as *cKp* transgenic mice) after mating with *Kras*‐G12D mutant allele and the allele point mutation of *p53* (R172H) under tamoxifen induction.

Through gene targeting technology based on the principle of homologous recombination, CAG promoter, loxp‐Stop‐loxp regulatory element and EGE‐PNKD‐CDS‐IRES‐EGFP‐WPRE‐pA expression element were inserted into Rosa26 site, and then Cas9 protein/sgRNA was injected into the zygote to construct the mutant, so that the gene was knocked in the mice (Figure S1A, Supporting Information).

Through the principle of homologous recombination, the locus of X‐over P1 derived from bacteriophage P1 was inserted into the key exons of the *PNKD* gene in mouse embryonic stem cells (ES), thereby knocking out the *PNKD* gene (Figure S1B, Supporting Information).

**Figure 2:**
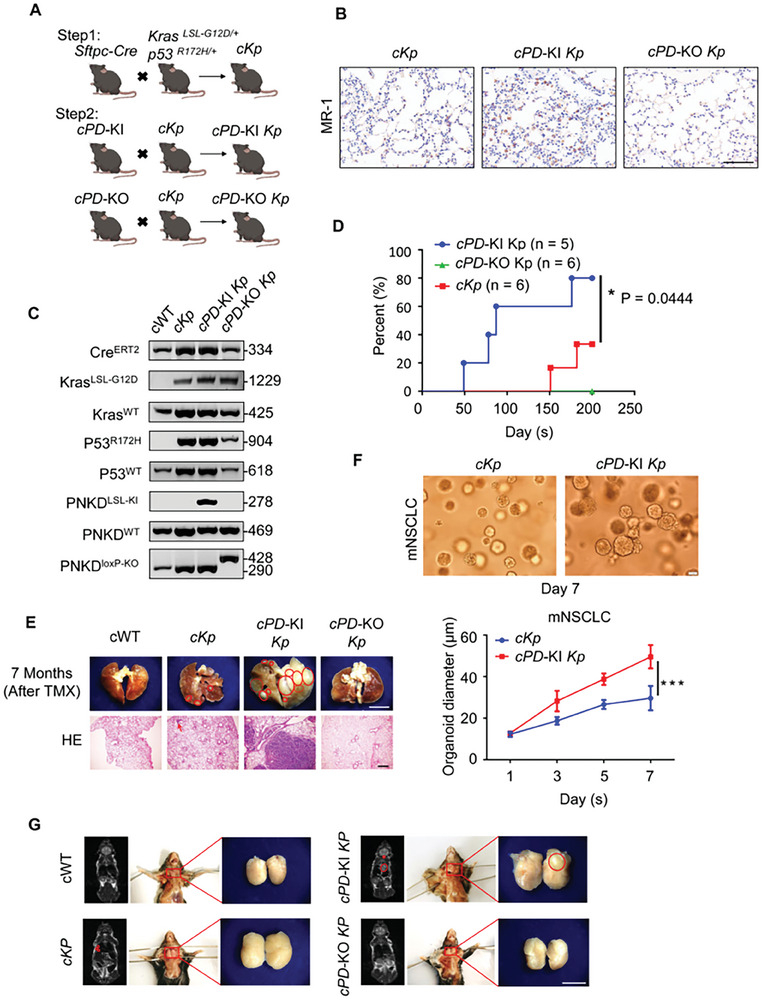
MR‐1 promotes the occurrence and development of NSCLC in transgenic mice. A) A schematic of the strategy for generating *sftpc‐Cre*/+; *Kras LSL‐G12D*/+; p*53 R172H*/+ mice (*cKp*), *sftpc‐Cre*/+; *Kras LSL‐G12D*/+; *p53 R172H*/+; *PNKD*‐KI mice (*cPD‐*KI *Kp*) and *sftpc‐Cre*/+; *Kras LSL‐G12D*/+; *p53 R172H*/+; *PNKD*‐KO mice (*cPD‐*KO *Kp*). B) IHC of MR‐1 in lung tissues of transgenic mice with the indicated genotypes. Scale bar, 100 µm. C) Confirming the indicated genotypes of transgenic mice through PCR. D) The incidence curve analysis of lung cancer for transgenic mice with the indicated genotypes at the indicated ages. Log‐rank (Mantel‐Cox) test. *p < 0.05. E) Seven months after tamoxifen induced disease in transgenic mice, representative images and hematoxylin‐eosin (HE) staining images of lung cancer. Scale bar, 500 µm. F) Images of lung cancer organoid derived from transgenic mice. Data are three independent means of measuring ± SEM. The statistical significance between the two groups was determined using a double tailed student *t*‐test. Scale bar, 20 µm. ***p < 0.005. G) Seven months after tamoxifen induced disease in transgenic mice, magnetic resonance imaging (MRI) was used for the whole body scanning of mice in vivo. Scale bar, 500 µm.

The above two types of transgenic mice were mated with wild‐type C57BL/6N mice to obtain *PNKD* transgenic mice with genotype stable overexpression/knockout, and mated with *c‐*transgenic mice to obtain transgenic mice with simultaneous overexpression of *PNKD* (*cPD*‐KI)/knockout of *PNKD* (*cPD*‐KO) of genes (Figure S1A,B, Supporting Information). Subsequently, *cPD*‐KI/*cPD*‐KO transgenic mice were mated with *cKp* transgenic mice to obtained *cPD*‐KO *Kp* and *cPD*‐KI *Kp* transgenic mice (**Figure** **2**A). The disease was induced by tamoxifen (75 mg kg^−1^) at 6 weeks of age. We validated the successful construction of transgenic mice by immunohistochemistry and PCR (Figure 2B,C). Two months after administration, we started to take micro‐CT scan of in‐vivo mouse lung monthly by InSyTe FLECT/CTTM system, and found that *PNKD* can accelerate the occurrence, development, and death of lung cancer, while knocking out *PNKD* does not cause disease (Figure 2D,E; Figure S2A, Supporting Information). We also detected the expression of Epithelial‐mesenchymal transition (EMT) related proteins in the tumor by Western Blotting, and found that MR‐1 can promote EMT (Figure S2B, Supporting Information). Seven months after administration, we found that c*PD*‐KI *Kp* transgenic mice had thyroid metastatic through Magnetic Resonance Imaging (MRI) scans (Figure 2G). CK8 is cytokeratin 8 (CK8), associated with increased invasiveness of the tumor in vitro and in vivo, and is preferentially expressed in NSCLC.^[^ ^42^ ^]^ We found that compared to *cKp* lung tumor tissue, *cPD*‐KI *Kp* lung tumor tissue and metastatic thyroid tissue showed higher expression of lung metastasis associated protein CK8 through immunohistochemistry (Figure S2C, Supporting Information). Invasion is a fundamental step in the progression of tumors toward metastasis. In order to investigate its invasiveness, we cultured organoids of mouse tumor tissue in a 3D invasion matrix and found that compared to control organoids, the *cPD*‐KI *Kp* group had better formation ability and exhibited stronger invasion and proliferation ability (Figure 2F). It may indicate that the expression of *PNKD* is sufficient and necessary for NSCLC organoids to show potential for metastasis and invasion. The above data suggests that *PNKD* may be crucial for the occurrence, development, and especially metastasis of lung cancer.

### 2.3 The Overexpression of MR‐1 Promoted the Metastasis of NSCLC Cells

**Figure 3:**
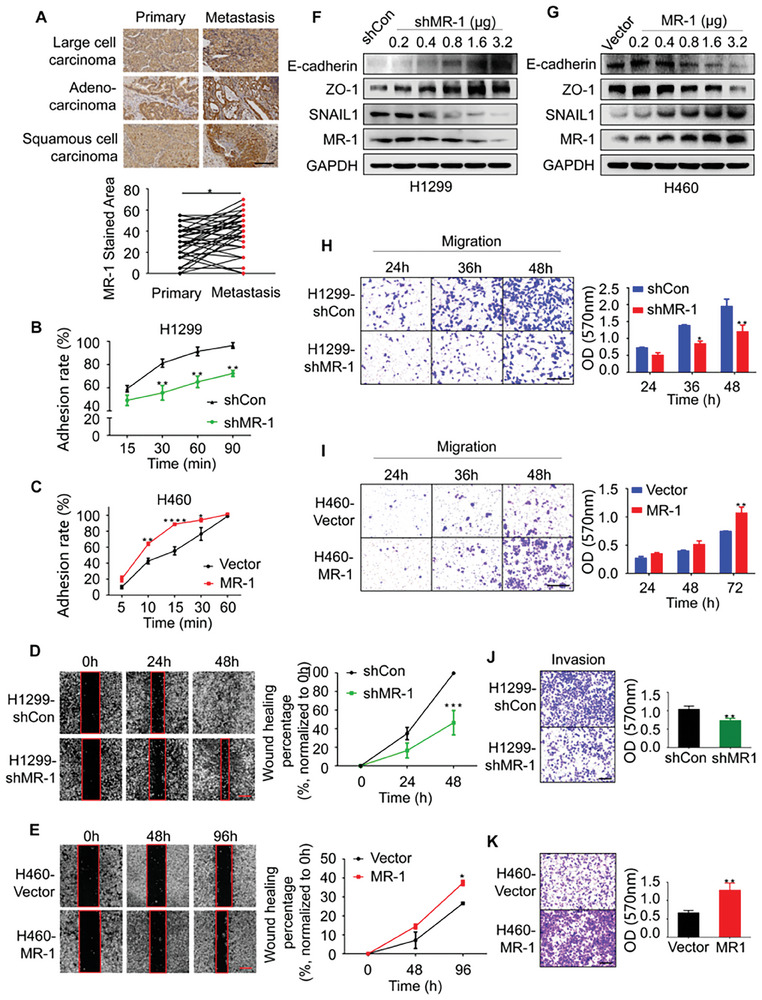
MR‐1 is essential for metastasis of NSCLC. A) IHC determined MR‐1 expression in NSCLC primary cancer tissue and its corresponding metastatic tissues. Scale bar, 200 µm. B,C) The changes of cell adhesion in H1299 and H460 cells with knockdown or overexpression of MR‐1. Cell activity was detected by CCK8 assay. A dose‐dependent curve was plotted using GraphPad Prism6 software. The data was shown as mean ± SEM (*n* = 3). Two‐way ANOVA. D,E) Representative images of wound healing experiments were performed to detect cell migration in H1299 and H460 cells with knockdown or overexpression of MR‐1. The cells migration rate = Migration area / initial area ×100%. Scale bar, 500 µm. Two‐way ANOVA. F,G) Western blot analysis of EMT related proteins in H1299 and H460 cells with knockdown or overexpression of MR‐1. H,I) Representative images of transwell experiments were performed to detect cell migration in H1299 and H460 cells with knockdown or overexpression of MR‐1. Scale bar, 200 µm. The data was shown as mean ± SEM (*n* = 3). Two‐way ANOVA. J,K) Representative images of invasion within 48 h by matrix gel assay in H1299 and H460 cells with MR‐1 knockdown or overexpression. Scale bar, 600 µm. The data was shown as mean ± SEM (*n* = 3). Two‐way ANOVA. *p < 0.05; **p < 0.01; ***p < 0.005; ****p < 0.001.

In order to explore the role of MR‐1 in promoting the progression of NSCLC, we integrated the data of 1014 lung cancer patients with clinical information from TCGA database, grouped by the median of *PNKD*, and found that it was highly correlated with the regulation of actin cytoskeleton through GSEA enrichment analysis (NES = 1.64, *P* = 0.004, Figure S3A, Supporting Information). At the same time, we found that the high expression of *PNKD* was associated with distal metastasis through R software v4.0.3 ggplot2 package analysis (Figure S3B, Supporting Information). Through stratified survival curves, we also observed that the high expression of *PNKD* was not conducive to prognosis in 153 NSCLC patients with lymph node metastasis (Figure S3C, Supporting Information). Therefore, wound healing experiments were conducted on normal bronchial epithelial cells BEAS‐2B and seven types of NSCLC cells. We fitted the curve based on their 48‐hour scratch healing rate and relative expression of MR‐1 protein, and found a positive correlation between them (*r* = 0.833, *P* = 0.015, Figure S3D–F, Supporting Information). Subsequently, the IHC tests were performed to detect the expression of MR‐1 in 40 NSCLC patients and their tissue arrays of corresponding metastatic cancer (**Figure** **3**A) through TMA (LUM961), and found that MR‐1 was highly expressed in metastatic cancer tissues. Therefore, we speculate that MR‐1 is highly correlated with NSCLC metastasis.

Based on the above results, we conducted in vitro experiments. H1299 and H460 cells were respectively selected as representative cell lines with strong metastasis, high MR‐1 expression and poor metastasis, low MR‐1 expression. The knockdown of MR‐1 expression in H1299 cells by transient transfection could weaken cell adhesion and scratch healing ability, inhibit the EMT process (epithelial markers E‐cadherin and ZO‐1 are negatively correlated with MR‐1, while stromal markers SNAIL1 are positively correlated with MR‐1), and reduce cell migration and invasion ability, while it was opposite to the overexpression of MR‐1 in H460 cells. (Figure 3B–K). Colony formation assay showed that knocking down MR‐1 inhibits cell proliferation in H1299 and A549 cells. We also performed wound healing experiments and detected the expression of EMT related proteins (Figure S4A–C, Supporting Information) on the representative NSCLC A549 cells to confirm the effect of MR‐1, and the results indicated that the knockdown of MR‐1 could inhibit the migration ability of A549 cells. The above data indicate that MR‐1 has a certain promoting effect on NSCLC metastasis.

### 2.4 MR‐1 Exerted Pro‐Metastatic Activity Through Notch3‐ICD

**Figure 4:**
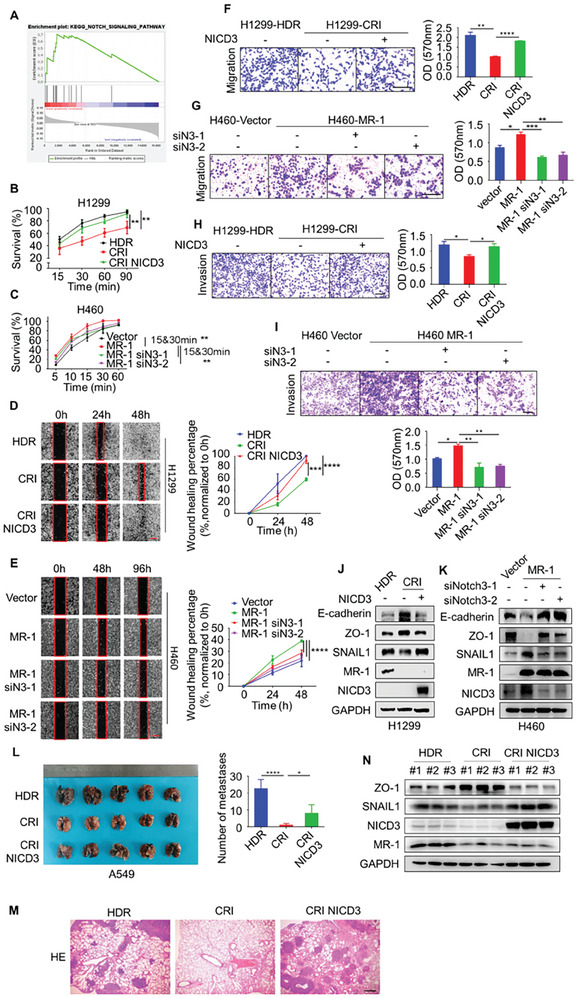
MR‐1 promotes NSCLC metastasis by regulating NICD3. A) The GSEA analysis of gene expression profiles of NSCLC patients with or without metastasis in the TCGA lung cancer datasets. (B‐K) Overexpressing or Silencing of NICD3 by transient transfection with pCDNA3.1‐HA‐NICD3/siNotch3 reversed cell adhesion ability B,C), cell scratch healing ability D,E Scale bar, 500 µm.), cell migration ability (F, G Scale bar, 200 µm.) cell invasion ability (H, I Scale bar, 600 µm.) and EMT related proteins expression J,K) in stable MR‐1‐knockdown or MR‐1‐overexpression H1299 and H460 cells. The data are shown as mean ± SEM (*n* = 3). Two‐way ANOVA. *p < 0.05; **p < 0.01; ***p < 0.005; ****p < 0.001. L) A549 cells (1.5×10^7^ cells) stabilized MR‐1‐knockdown or together with NICD3 overexpression were intravenously injected into nude mice (*n* = 5 per group), the numbers of metastatic foci of indicated lung tissue, representative images of HE staining (M Scale bar, 400 µm.) and the expression of EMT related proteins (N) are shown. The data are shown as mean ± SEM (*n* = 3). Two‐way ANOVA. *p < 0.05; ****p < 0.001.

To investigate whether MR‐1 regulates the metastasis of lung cancer cells, we profiled RNA sequencing (RNA seq) analysis to identify different gene expression patterns in control and MR‐1‐knockdown H1299 cells. Subsequently, KEGG pathway enrichment analysis was visualized as a bubble plot (Figure S5A, Supporting Information) and combined with GSEA enrichment analysis to group 1014 NSCLC patients in the TCGA database based on whether metastasis occurred, which showed that both can enrich the Notch signaling pathway (NES = 1.62, *P* = 0.012, **Figure** **4**A). Based on this enrichment result, we constructed H1299 cells with stably MR‐1‐knockdown by CRISPR/Cas9 technology and H460 cells with stably MR‐1‐overexpression (Figure S5C, Supporting Information). The expression of MR‐1 was positively correlated with the mRNA levels of target genes HES1, HEY1, and HEY2 of the Notch signaling pathway through PCR assays (Figure S5B, Supporting Information).

Notch signaling pathway receptors are Notch1‐4 transmembrane glycoproteins.^[^ ^43^ ^]^ The receptor protein undergoes three segment cleavage to release intracellular domains (ICDs) that translocate into the nucleus to regulate the transcription of target genes and exert cancer promoting activity. In addition, since Notch4 is mainly expressed in placental blood vessels and other endothelial cells, we detected the changes of Notch1‐3‐ICD (abbreviated as NICD1‐3) protein levels by the stable cells constructed above and determined that MR‐1 only affected the expression of NICD3 (Figure S5C,D, Supporting Information). Due to its cancer promoting activity generated after translocation into the nucleus, we conducted cytoplasmic‐nuclear separation experiments, and found that the nuclear localization of NICD3 was decreased in H1299 cells with stably MR‐1‐knockdown, while H460 cells with stably MR‐1‐overexpression promoted the nuclear localization of NICD3 (Figure S5E, Supporting Information).

Based on the above results, we further investigated whether MR‐1 exerted its metastasis promoting activity in lung cancer through NICD3. We found that the overexpression of NICD3 in H1299 cells with stably MR‐1‐knockdown could decrease the increased expression of EMT epithelial markers E‐cadherin and ZO‐1, the decreased expression of stromal marker SNAIL1, adhesion, migration and invasion ability, while the knockdown of NICD3 in H460 cells with stably MR‐1‐ overexpression led to the opposite results (Figure 4B‐K). It is worth noting that the overexpression of Notch signal target gene HES1 while the knockdown of MR‐1, we also found that the migration ability and EMT transformation were decreased caused by the knockdown of MR‐1 through wound healing experiments and EMT protein detection (Figure S6A,B, Supporting Information). This further confirms that MR‐1 may exert its metastasis promoting activity through the Notch signaling pathway. In addition, after the overexpression of NICD3 in stably MR‐1‐knockdown A549 cells, we conducted in vivo experiments on a nude mouse tail vein injection lung metastasis model, and found that NICD3 could reverse the formation of decreased lung metastases caused by MR‐1‐knockdown (Figure 4L, M). We also validated the expression of EMT related proteins in lung tissue by Western Blot (Figure 4N), which is consistent with the above in vitro results. These data indicate that NICD3 is crucial for the metastasis of MR‐1 in NSCLC cells.

### 2.5 MR‐1 Enhanced the Stability of Notch3‐ICD by Inhibiting Degradation of Ubiquitin Lysosome Pathway

**Figure 5:**
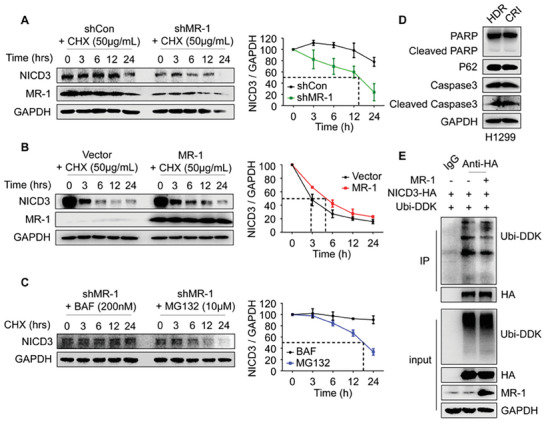
MR‐1 stabilizes NICD3 by inhibiting ubiquitin lysosomal degradation pathway. A) H1299 cells were treated in CHX with or without MR‐1‐knockdown for the indicated time, and the NICD3 protein was detected by Western blotting. The experiment was repeated three times and the representative image were shown. The quantity analysis of protein density analysis normalized to GAPDH. B) HEK‐293T cells were treated in CHX with or without MR‐1‐overexpression for the indicated time, and the NICD3 protein was detected by Western blotting. The experiment was repeated three times and the representative image were shown. The quantity analysis of protein density analysis normalized to GAPDH. C) H1299 cells with MR‐1‐knockdown were treated with combination of CHX and MG132 or CHX and BAF was added for indicated time, and NICD3 protein was detected by Western blotting. The experiment was repeated three times and the representative image were shown. The quantity analysis of protein density analysis normalized to GAPDH. D) Western blot analysis of the autophagy marker (p62) and apoptosis markers (cleaved PARP and cleaved caspase3) in stable MR‐1‐ knockdown H1299 cells. E) The effect of MR‐1 on NICD3 ubiquitination. HEK‐293T cells were transfected with NICD3‐HA, ubiquitin‐DDK, or MR‐1 expression plasmid for 48 h and then treated with BAF (200 nmol L^−1^). Cell extracts were IP with an anti‐HA Ab. Ubiquitinated NICD3 was detected by Western blot. The data are shown as mean ± SEM (*n* = 3).

The regulation of protein levels is generally achieved through two aspects: transcriptional‐level control and protein stability regulation. The Notch signaling pathway induces the sequential cleavage of Notch receptors through receptor ligand interactions, ultimately leading to the release of NICD in signal‐receiving cells.^[^ ^16^, ^17^, ^18^ ^]^ As shown in Figure S5D (Supporting Information), we did not observe the significant changes of Notch receptors in different cleavage forms regulated by MR‐1. However, MR‐1 has a significant impact on the protein level of NICD3, so we speculate that MR‐1 may affect the stability of NICD3 in the cytoplasm. We found that the knockdown of MR‐1 could significantly accelerate the degradation of NICD3 in H1299 cells, with a reduced half‐life from over 24 h to about 15 h by inhibiting protein synthesis through cycloheximide (CHX). The overexpression of MR‐1 increases the half‐life of NICD3 degradation by ≈2 h (**Figure** **5**A,B). Further experiments found that while the knockdown of MR‐1, the degradation of NICD3 was inhibited by lysosomal inhibitor bafenomycin (BAF), but not proteasome inhibitor MG132 (Figure 5C). The classic lysosomal pathway is often associated with autophagy and apoptosis, but in stable MR‐1‐ knockdown H1299 cells, it did not cause the changes of autophagy markers (p62) and apoptosis markers (PARP and caspase3) through Western blot. The degradation mode of NICD3 is different from that of NICD1 and NICD2,^[^ ^44^, ^45^, ^46^ ^]^ which may be related to the number and position of lysine residues in the NICD3 sequence.^[^ ^47^ ^]^ We conducted sequence alignment of NICD1‐3 through Cluster X2.1, which was visualized by GeneDoc, and found that only 21 lysine residues were present in the NICD3 sequence, and compared to NICD1 and 2, there were no lysine dense regions (Figure S7, Supporting Information, 400–450). The above findings may be the main reason for the low sensitivity to ubiquitination and the occurrence of mono‐ubiquitination modification of NICD3. Subsequently, we found that the overexpression of MR‐1 could reduce the mono‐ubiquitination of NICD3 through Co‐IP experiments (Figure 5E). These data indicate that MR‐1 interferes with the degradation of NICD3 through mono‐ ubiquitin‐lysosome system.

### 2.6 MR‐1 Affected the Ubiquitination of NICD3 Through E3 Ubiquitin Ligase ITCH

**Figure 6:**
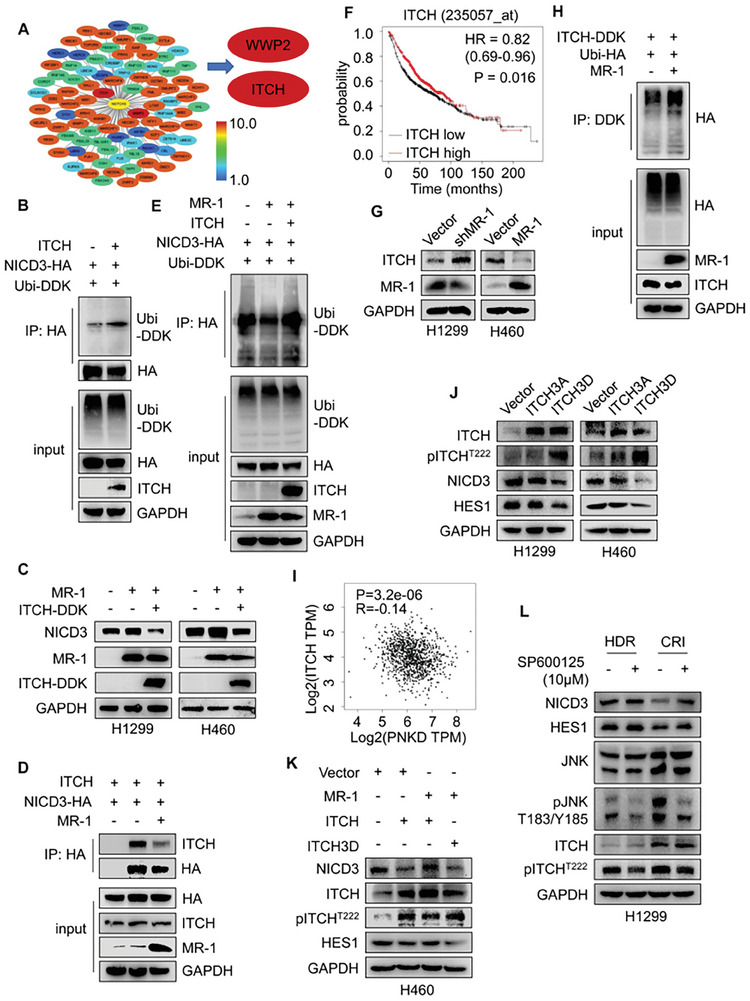
MR‐1 stabilizes NICD3 by inhibiting ITCH mediated down regulation of NICD3 ubiquitination modification. A) Predicting Specific E3 Ubiquitin Ligase with Notch3 as Substrate through Ubibrowser 2.0. ITCH and WWP2 with the highest coefficients were selected for subsequent experiments. B) pCDNA3.1‐HA‐NICD3, pCMV6‐ITCH and pCMV3‐DDK ubiquitin plasmids were co‐transfected into HEK‐293T cells for 48 h, and the cell extracts were IP with anti‐HA Ab. Ubiquitinated NICD3 was detected by Western blot. C) After transient transfection of pCDNA3.1‐MR‐1, pCMV6‐DDK‐ITCH plasmids in H1299 and H460 cells for 48 h, the effects of MR‐1 and ITCH on NICD3 expression were detected by Western blotting. D) HEK‐293T cells were transfected with the indicated plasmids for 48 h. Cell extracts were IP with an anti‐HA Ab. The interaction between ITCH and NICD3 was detected by Western blot. E) HEK‐293T cells were transfected with the indicated plasmids for 48 h. Cell extracts were IP with an anti‐HA Ab. Ubiquitinated NICD3 was detected by Western blot. F) OS of lung cancer patients grouped by ITCH expression level. Data was analyzed by Kaplan‐Meier plotter. G) The changes of the expression of ITCH in H1299 and H460 cells with knockdown or overexpression of MR‐1. H) pCDNA3.1‐MR‐1, pCMV6‐DDK‐ITCH and pCMV3‐HA ubiquitin plasmids were co transfected into HEK‐293T cells for 48 h, and the cell extracts were IP with anti‐DDK Ab. Ubiquitinated ITCH was detected by Western blot. I) PNKD and ITCH expression levels were negatively correlated in the GEPIA database. J) pCMV6‐DDK‐ITCH S199A, T222A, S232A (ITCH‐3A) and S199D, T222D, S232D (ITCH‐3D) mutant expression plasmids were constructed. After transient transfection of the above plasmids in HEK‐293T cells for 48 h, the expression of related proteins were detected by Western blot. K) After transient transfection of MR‐1 with ITCH or ITCH‐3D in H460 cells, the expression of related proteins was detected by Western blotting. L) JNK inhibitor (SP600125) was used for 1 h treatment in stable MR‐1‐knockdown H1299 cells and the expression of related proteins was detected by Western blot.

E3 ubiquitin ligase can specifically recognize and bind to substrate proteins in the ubiquitination process. We predicted the potential E3 ubiquitin ligase in Notch3 by Ubibrowser, which was visualized by Cytoscape_V3.9.1. We selected two known E3 ubiquitin ligases with the highest correlation coefficients for the experiment, E3 ubiquitin‐protein ligase Itchy homolog (ITCH, also known as AIP4) and WW domain‐containing protein 2 (WWP2) (**Figure** **6**A).^[^ ^48^, ^49^ ^]^ To confirm that the both are E3 ubiquitin ligases of NICD3, we performed further experiments of Co‐IP and Western Blot, and found that both WWP2 and ITCH could increase the ubiquitination level of NICD3 (Figure 6B; Figure S8A, Supporting Information), and reverse the increased expression of NICD3 caused by the overexpression of MR‐1 (Figure 6C; Figure S8B, Supporting Information). We conducted Co‐IP experiments to verify whether the two are key nodes of MR‐1‐mediated the regulation of NICD3 stability and found that MR‐1 could not affect the interaction between WWP2 and NICD3 (Figure S8C, Supporting Information), but could affect the interaction between ITCH and NICD3. ITCH could also reverse the decreased ubiquitination level of NICD3 caused by MR‐1 (Figure 6D, E). In addition, Kaplan‐Meier plot analysis of clinical data from 1144 NSCLC patients demonstrated that ITCH (235057_at) is beneficial for their prognosis (Figure 6F). It is worth noting that MR‐1 can negatively regulate ITCH, resulting in the increase of it‐self ubiquitination level (Figure 6G, H). We also found a negative correlation between *PNKD* and *ITCH* through the GEPIA database (R = −0.14, *P* = 3.2×10^−6^, Figure 6I).

It was reported^[^ ^50^ ^]^ that the E3 ubiquitin ligase activity of ITCH is usually affected by phosphorylation at its three sites, S199, T222, and S232. To confirm whether the phosphorylation at the three sites of ITCH is necessary for the ubiquitination of NICD3, we constructed ITCH3A (S199A, T222A, S232A, inhibited phosphorylation) and ITCH3D (S199D, T222D, S232D, sustained phosphorylation) mutant plasmids. We verified the necessity of ITCH phosphorylation in the regulation of NICD3 through Western Blot (Figure 6J). According to previous reports, the phosphorylation at the above three sites of ITCH requires the involvement of JNK kinases. After the knockdown of MR‐1 and the addition of SP600125 (JNK inhibitor), we found that the increased level of ITCH phosphorylation caused by the knockdown of MR‐1 was reduced, resulting in a decrease in the inhibitory effect of ITCH on NICD3 and an increase in the expressions of NICD3 and HES1 (Figure 6K). The ubiquitination of ITCH3D on NICD3 could not be reversed by MR‐1 (Figure 6L).

In summary, MR‐1 can affect the level of ITCH protein indirectly through JNK or directly, thereby affect the ubiquitination of NICD3 and regulate the level of its protein and the level of target gene proteins caused by entry into the nucleus.

### 2.7 Interference with the Effect of MR‐1 on NICD3 Alleviated Lung Cancer Metastasis

**Figure 7:**
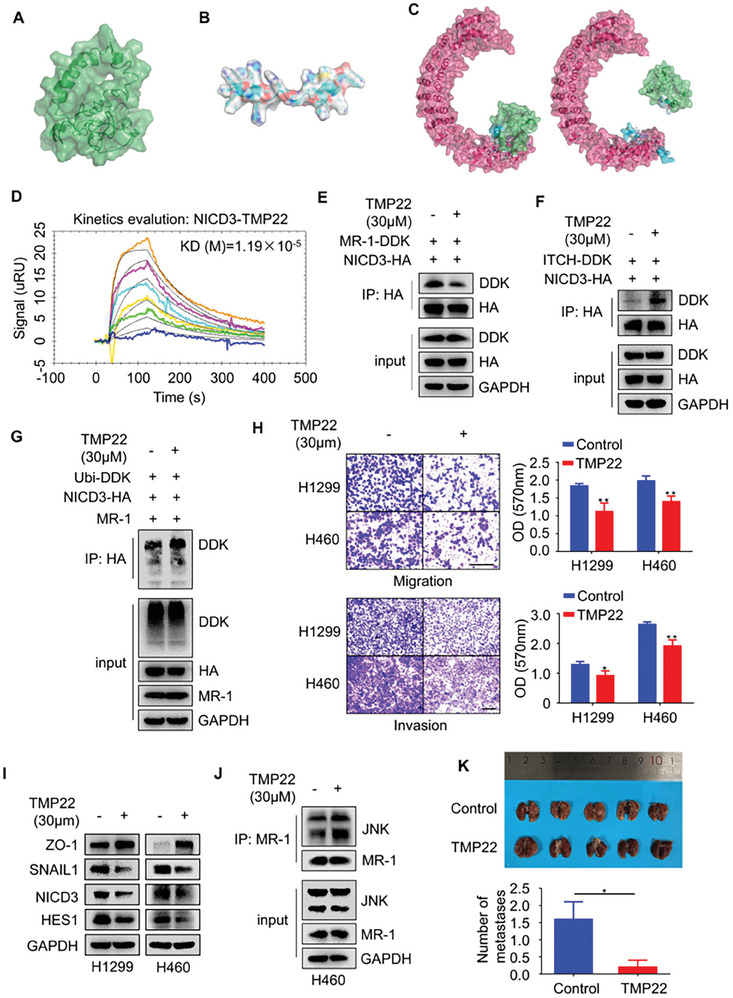
TMP22 affects the interaction of MR‐1 and NICD3. A) The prediction of MR‐1 3D structure and schematic structure through I‐TASSER server and AlphaFold v2.3.2. B) The prediction of peptides TMP22 3D structure and schematic structure through I‐TASSER server and AlphaFold v2.3.2. C) The prediction of peptides NICD3 3D structure and schematic structure through I‐TASSER server and AlphaFold v2.3.2. The structure‐Based Design of MR‐1 or TMP22 against NICD3 through HDOCK. D) SPR binding curve of TMP22 with NICD3 (double dilute from 100 µM to 1.57 µM). E) HEK‐293T cells were transfected with pCDNA3.1‐HA‐NICD3 and pCMV6‐DDK‐MR‐1 plasmids for 24 h and treated with TMP22 for 24 h. Cell extracts were IP with an anti‐HA Ab. The interaction between MR‐1 and NICD3 was detected by Western blot. F) HEK‐293T cells were transfected with pCDNA3.1‐HA‐NICD3 and pCMV6‐DDK‐ITCH plasmids for 24 h and treated with TMP22 for 24 h. Cell extracts were IP with an anti‐HA Ab. The interaction between ITCH and NICD3 was detected by Western blot. G) HEK‐293T cells were transfected with pCDNA3.1‐HA‐NICD3, pCDNA3.1‐MR‐1 and pCMV3‐DDK ubiquitin plasmids for 24 h and treated with TMP22 for 24 h. Cell extracts were IP with an anti‐HA Ab. The interaction between ubiquitin and NICD3 was detected by immunoblotting. H) The representative images of transwell experiments within 48 h and invasion within 48 h by matrix gel assay were performed to detect cell migration and invasion in H1299 and H460 cells with 48 h treatment of TMP22. Scale bar, 200 µm. The data was shown as mean ± SEM (*n* = 3). Two‐way ANOVA. *p << 0.05; **p < 0.01. I) Western blot analysis of EMT related proteins in H1299 and H460 cells with 24 h treatment of TMP22. J) After 24 h treatment with TMP22, endogenous binding of JNK and NICD3 was detected in H460 cells by Western blot. (K) A549 cells (1×10^7^ cells) were injected intravenously into nude mice (*n* = 5 per group), followed by daily intravenous injection of TMP22 (50 mg kg^−1^) five days later. The representative image shows the number of lung tissue metastases observed on the 21st day.

In order to verify the key role of MR‐1 for NICD3 in lung cancer metastasis, we attempted to design and screen short peptides (α Spiral peptides) that could inhibit this protein‐protein interaction (PPI) to interrupt this regulatory effect.^[^ ^51^ ^]^ After preliminary confirmation of the interaction between MR‐1 and NICD3 (Figure S9A, Supporting Information), we truncated MR‐1. According to the results of Predict protein (Figure S9B, Supporting Information) and TMHMM (Figure S9C, Supporting Information) online analysis tools, MR‐1 could be a transmembrane protein, with transmembrane domains ranging from 75 to 92 amino acids. To prevent truncation of the transmembrane domains, we expanded the predicted domains to 68 to 107 amino acid. Based on this, we constructed different truncated plasmids: 1–67, 68–142, 1–107, and 108–142. Each base sequence was cloned into the pCMV6‐AC‐DDK vector to construct a truncated plasmid. The sequencing results were compared with the NCBI database by BLAST tool, indicating the successful construction of plasmid. However, after the 293T transfection experiment, it was found that only 68–142 and 1–107 plasmids could be expressed through Western blot (Figure S9D, E, Supporting Information). And these two plasmids were used for subsequent experiments. Based on the experimental results of Co‐IP, we speculated that the domain of interaction between MR‐1 and NICD3 was 68 to 107 amino acids (Figure S9F, Supporting Information). We also found that there is a certain interaction between MR‐1 and ITCH, and the interaction region may be 68 to 107 amino acids (Figure S9G,H, Supporting Information). We constructed the 68–107 fragment‐deficient MR‐1 plasmid and found that MR‐1 with the fragment deletion no longer interacts with NICD3 (Figure S10A, Supporting Information). In addition, it no longer has inhibitory effect on protein expression and phosphorylation levels of ITCH, and stimulative effect on the EMT process and the ability of cells migration and invasion (Figure S10B, C, Supporting Information). We further predicted the spatial structure of MR‐1 (**Figure** **7**A), and speculated that the interaction region between MR‐1 and NICD3 was α Spiral region in the cytoplasm near the cell membrane in 68 to 107 amino acids. TMP22 (a fusion peptide with TAT linking to MR‐1 Protein legion of 22 amino acids) was designed and screened as an active fusion peptide that could competitively bind to NICD3 (Figure 7B,C; Figure S11, Supporting Information).

The protein‐protein docking analysis was implemented using the HDOCK webserver, which used a combination of template‐based and ab initio method to incorporate the binding interface information into traditional global docking.^[^ ^52^ ^]^ The NICD3 protein was defined as receptor molecule due to its longer sequence, while MR‐1 and TMP22 were input as ligand molecule respectively. And other parameters were set to the default. They were further analyzed in the Pymol program. Although the docking score should not be treated as the true binding affinity of two molecules, a more negative score usually means a more possible binding model. A confidence score above 0.7 means the two molecules would be very likely to bind (Figure 7C). Subsequently, based on the SPR validation in Figure 7D, we found that the Ka, Kd, and KD of NICD3 protein (The results of purification are shown in Figure S12, Supporting Information) and TMP22 were 6.87×10^2^ M^−1^s^−1^, 8.15×10^−3^ s^−1^, and 1.19×10^−5^ M. This result indicates that NICD3 and TMP22 can be directly combined and the equilibrium dissociation constant (KD) of both is 1.19×10^−5^ M (Kd/Ka), further verifying the docking results. Then we plan to explore the effect of TMP22 on the process of MR‐1‐mediated the regulation of NICD3 and lung cancer metastasis.

The experimental results of Co‐IP showed that TMP22 effectively disrupted the interaction between MR‐1 and NICD3 and moderately reduced the expression of NICD3 and downstream molecule HES1 (Figure 7E, I). In addition, TMP22 could also increase the interaction between ITCH and NICD3, and accelerate the level of NICD3 ubiquitination (Figure 7F, G). Furthermore, we conducted transwell and matrigel invasion assays in H1299 and H460 cells and found that by the action of TMP22, the ability of cell migration and invasion was decreased. Similarly, TMP22 also inhibited the EMT process of H1299 and H460 cells (Figure 7H, I). However, the inhibitory effect of TMP22 on the colony formation ability is not very significant (Figure S13, Supporting Information). TMP22 might be more effective in the inhibiting metastasis. Interestingly, we also observed that TMP22 could promote the interaction between MR‐1 and JNK (Figure 7J), which indirectly confirms that MR‐1 might affect the activity of ITCH through JNK, leading to the degradation of NCID3. We also observed the inhibitory effect of TMP22 on the development of lung cancer in vivo experiments (Figure 7K). Due to our reduction in cell count and prolongation of the experimental cycle, we found liver metastases in the control group (80%), while TMP22 significantly inhibited the formation of liver metastases (Figure S14, Supporting Information). These data confirm that interrupting the interaction between MR‐1 and NICD3 may be a promising treatment strategy for lung cancer.

## 3 Discussion

In this study, we selected the shortest splicing form of MR‐1, MR‐1S (NM‐001077399.1), which is the only one with cancer effects.^[^ ^8^ ^]^ It consists of three different exons and encodes a 15.6 kDa protein with 142 amino acids (aa), without homology to any known protein.^[^ ^53^ ^]^ Clinical data shows that MR‐1 is overexpressed in gastric cancer and pancreatic ductal adenocarcinoma, and is significantly correlated with clinical staging and poor survival rate.^[^ ^11^, ^13^ ^]^ Preliminary laboratory studies focused on liver cancer and breast cancer. MR‐1 can promote cell proliferation and migration of human liver cancer and breast cancer cells.^[^ ^14^, ^15^ ^]^ Consistent with the above results, bioinformatics analysis showed that MR‐1 is overexpressed in NSCLC, which was also verified through in vitro experiments. Its overexpression was positively correlated with poor prognosis and has certain diagnostic efficacy. NSCLC has poor response to chemotherapy and delayed detection, so although clinical treatment methods for cancer have become increasingly diverse, the 5‐year survival rate is still below 20%.^[^ ^5^ ^]^

*Cre/ERT2* mice are a type of mice that contain ligand binding region mutants (ERT) of estrogen receptors and fusion proteins of *Cre* recombinase. *Cre/ERT2* is in an inactive state in the cytoplasm without tamoxifen induction. When Cre/ERT2 induced by tamoxifen, it can enter the nucleus to exert *Cre* recombinase activity. By using different promoters, *Cre* enzyme expression can be specifically activated in different tissues or cells. The *sftpc Cre/ERT2* knock‐in mice used in this paper carry the IRES cre sequence in the 3′ ‐ UTR region of the *sftpc* gene, and express Cre protein in type II alveolar tissue epithelial cells induced by tamoxifen. This strain exhibits high lung tissue specificity in the Cre/loxp system. For combined overexpression of PNKD in mice (cPD‐KI Kp mice), we found that MR‐1 can not only accelerate the progression and death of NSCLC, but also enable metastasis, while *cPD*‐KO *Kp* mice does not suffer from cancer. Due to overexpression of *PNKD*, transgenic mice with lung cancer developed rapidly and had a short survival period, making it difficult to collect metastasis related data. To this end, we conducted immunohistochemistry experiments to detect lung cancer metastasis related indicator CK8,^[^ ^42^ ^]^ Western Blot to detect EMT related indicators, and organoid experiments. All results of above experiments indicated that primary lung cancer caused by *cPD*‐KI *Kp* genotype has a stronger trend of metastasis and invasion. Therefore, it suggests that MR‐1 may become a more effective treatment method to curb the malignant progression of NSCLC and improve its survival rate.

We conducted enrichment analysis on MR‐1 expression and whether it was transferred or not by grouping them separately, and found that both were able to enrich the Notch signaling pathway. The Notch signaling pathway is mainly composed of four receptors. Notch1 and Notch2 are widely expressed throughout the entire developmental process in many tissues and adult mammals.^[^ ^54^ ^]^ By contrast, Notch3 is most abundant in vascular smooth muscle and pericytes.^[^ ^55^ ^]^ Notch4 is mainly expressed in endothelial cells.^[^ ^43^ ^]^ After cleavage, the Notch receptor enters the nucleus and binds to the transcription factor CSL, forming the NICD/CSL transcriptional activation complex, thereby activating the target genes of the basic helix loop helix (bHLH) family of transcription repressors such as HES and HEY to exert biological effects. Our study indicates that MR‐1 exerts pro‐metastatic activity through NICD3 . Overexpression of target gene HES1 in the Notch signaling pathway confirms activation of the pathway, and can reverse the decreased metastatic ability caused by knocking down MR‐1.

We found that NICD3 has fewer lysine residues and does not have a dense lysine region through amino acid sequence alignment of NICD1‐3, indicating its lower sensitivity to E3 ubiquitin ligase, which is also one of the possible reasons for the occurrence of mono‐ubiquitination modification of NICD3. Poly‐ubiquitination modification is usually associated with degradation of substrate proteasome pathway. Therefore, NICD3 may not be associated to the proteasome degradation pathway. The conclusion of this article is consistent with previous reports which have shown that^[^ ^56^ ^]^ lysosomal inhibition can delay the degradation of NICD3. We excluded the classical lysosomal pathway by detecting autophagy and apoptosis markers. We also observed the presence of PPxY motifs in the gene sequence of NICD3 through the amino acid sequence alignment of NICD1‐3 (Figure S7, Supporting Information, 670–680). This motif is a specific amino acid motif required for specific recognition between the HECTs family NEDD4‐like E3 ubiquitin ligases and substrate.^[^ ^57^, ^58^ ^]^ WWP2 and ITCH confirmed as Notch3 E3 ubiquitin ligases in this study are both NEDD4‐like E3 ubiquitin ligases,^[^ ^49^, ^57^ ^]^ but MR‐1 only affects the interaction between ITCH and NICD3. In this study, we found that MR‐1 interacts with NICD3 and affects the expression of NICD3. Moreover, MR‐1 can inhibit the interaction between ITCH and NICD3, the mono‐ubiquitin lysosomal degradation of NICD3, and thus promote the metastasis of NSCLC. Disrupting the interaction between MR‐1 and NICD3 effectively inhibits the metastasis of lung cancer cells and accelerates the degradation of NICD3 by ITCH. Due to the competitive relationship between MR‐1 and TMP22, the anti‐metastasis effect of TMP22 is not as significant as directly knocking down MR‐1. In addition, the inhibitory effect of TMP22 on the colony formation ability of lung cancer cells is not satisfactory, possibly because the interference effect caused by TMP22 mainly affects cell metastasis. The Notch signaling pathway requires adjacent cells to transmit signals through receptor ligand binding. The independent survival of cells in clone formation may also be one of the reasons why the effect of TMP22 is not significant.

ITCH, as an E3 ubiquitin ligase, has an isomeric self‐inhibitory effect and requires activator protein to fully achieve its function, thereby leading to substrate ubiquitination.^[^ ^59^ ^]^ The central region of ITCH containing PRR and WW motifs interacts with its HECT domain to inhibit the activity of the HECT domain. During phosphorylation of the S199, T222, and S232 sites of ITCH mediated by JNK, the intramolecular interaction between the central WW domain and the HECT domain of ITCH is decreased, and the structure of the WW domain is altered due to electrostatic repulsion. ITCH changes from a closed to an open conformation, allowing substrate access to its WW domains, which can enhance the catalytic activity of ITCH.^[^ ^50^, ^60^, ^61^ ^]^ Our study also found that MR‐1 has a regulatory effect on JNK, and may even have some kinase regulatory activity, which may also lead to conformation changes and self‐ubiquitination of ITCH.^[^ ^62^ ^]^ This is also the focus of our next work.

Previous reports have shown that^[^ ^31^, ^32^, ^33^, ^34^ ^]^ Notch3 has a certain promoting effect on the occurrence, development, and metastasis of lung cancer. With regard to E3 ubiquitin ligase ITCH of Notch3, research has shown that^[^ ^63^ ^]^ ITCH can regulate the degradation of the tumor suppressor gene p63 and its improper removal is also closely related to malignant transformation and chemical resistance.^[^ ^64^ ^]^ However, it has also been reported that^[^ ^59^ ^]^ the activation of ITCH can mediate the ubiquitination and endocytosis of the chemokine receptor CXCR4, thereby reducing the stemness of breast cancer cells. In addition, ITCH‐mediated ubiquitination can perform a tumor‐suppressive role via impaired degradation of WWOX,^[^ ^65^ ^]^ which may be due to the fact that most of the evidence accumulated to date on the role of ITCH in tumorigenesis has come from in vitro or in vitro models. Therefore, the lack of a complete tumor microenvironment may not be able to fully assess all the pathophysiological changes that ITCH may affect in cancer cells and at the systemic level. Targeting ITCH directly can also lead to the changes in protein levels of its various substrate of ubiquitination, which may bring out uncontrollable pathophysiological changes. It was reported that^[^ ^66^ ^]^ amot130 repurposes ITCH from its previously described role in degrading large tumor suppressor 1 to the inhibition of YAP and cell growth. We speculate that it may have a better and clearer therapeutic effect on cancer by regulating its upstream factors.

In recent years, research drug development targeting Notch signaling pathway has intensified. According to the signaling process, they are mainly classified into four categories.^[^ ^67^ ^]^ Biologics that bind to the extracellular region of Notch ligands or receptors; ADAM17 inhibitors that block the initial step of ligand‐induced Notch receptor processing; Small‐molecule γ‐secretase inhibitors that block the final step of ligand‐induced Notch receptor processing; Inhibitors of NICD protein‐protein interactions (PPI) that block NICD‐dependent transcription of Notch target genes. Among them, ADAM17 inhibitors were limited to combination studies in oncology. The γ‐secretase inhibitors RO4929097^[^ ^68^ ^]^ (Clinicaltrials.gov ID: NCT01116687, NCT01120275, NCT01175343 and NCT01232829), AL101^[^ ^69^ ^]^ (Clinicaltrials.gov ID: NCT01292655) and LY3039478^[^ ^70^ ^]^ (Clinicaltrials.gov ID: NCT01695005), and NICD PPI inhibitor CB‐103^[^ ^71^ ^]^ (Clinicaltrials.gov ID: NCT03422679), suffered from insufficient antitumor activity or a wide variety of side effects, probably due to insufficient specificity. In contrast, the fastest progressing is Notch ligand DLL3 ligand inhibitor, Rovalpituzumab tesirine, which has entered phase III clinical trials but has also been discontinued due to lack of efficacy.^[^ ^72^ ^]^ There are also Chimeric Antigen Receptor T‐Cell Immunotherapy (CAR‐T) that are in clinical studies, such as AMG119 (Clinicaltrials.gov ID: NCT03392064) and LB2102 (Clinicaltrials.gov ID: NCT05680922). Therefore, biologics that can selectively block Notch ligands or receptors have been predicted to be the most effective cancer therapies.^[^ ^67^ ^]^

In this article, MR‐1 indirectly exerts its cancer promoting effect by maintaining the stability of NICD3 in the cytoplasm by inhibiting ITCH. And MR‐1 is selective for NICD3. This mechanism provides relevant functional significance for MR‐1 to become a new target for the treatment of NSCLC and increases the possibility of MR‐1 as a drug‐forming target against the Notch signaling pathway. The MR‐1/ITCH/NICD3/HES1 axis may be a promising therapeutic target in the progression of lung cancer.

## 4 Conclusion

Our study suggests that MR‐1 increases the production of NICD3 in the cytoplasm, leading to increasing NICD3 into the nucleus, exerting the transcriptional activation, and promoting the metastasis of lung cancer cells. The interaction between MR‐1 and NICD3 stabilizes the intracellular NICD3 and inhibits its degradation. Interference with this interaction can inhibit the metastasis of lung cancer cells. And this inhibition of degradation is achieved through the effect of E3 ubiquitin ligase ITCH by MR‐1. Due to its unique structure and sequence, the degradation of NICD3 is achieved through the monoubiquitin lysosomal pathway. In summary, the interruption of MR‐1/ITCH/ NICD3/HES1 axis may be a new strategy for lung cancer treatment.

## 5 Experimental Section

### Ethics Approval

The collection of tissue samples was approved by the Ethics Committee of the First Hospital of China Medical University according to the Declaration of Helsinki, and all participants provided written informed consent with ethical approval number, [2021]387. All methods were performed in accordance with the relevant guidelines and regulations.

### Reagents

Transwell chambers pre‐coated with (354480) or without (3422) matrigel were purchased from Corning (Steuben County, New York, USA). A CCK‐8 reagent kit (FC101‐01) was purchased from TransGen Company (Beijing, China). Anti‐PARP, anti‐caspase 3, anti‐cleaved caspase 3, anti‐GAPDH, anti‐JNK, anti‐E‐cadherin, anti‐ZO‐1, anti‐SNAIL1, anti‐HES1, anti‐p62, anti‐HA, anti‐DDK and anti‐ubiquitin antibodies were purchased from Cell Signaling Technology (Danvers, MA, USA). Anti‐Notch1, anti‐Notch2, anti‐Notch3, anti‐phospho‐JNK and anti‐Lamin B1 were purchased from Beyotime (Jiangsu, China). Peroxidase‐conjugated goat antimouse and goat antirabbit secondary antibodies were purchased from ZSGQ‐BIO Company (Beijing, China). Anti‐ITCH antibody was purchased from Bioss (Woburn, MA, USA). Anti‐*PNKD*, Anti‐phospho‐ITCH antibodies, Cycloheximide (CHX) (01810), MG132 (C2211), and bafilomycin A1 (BAF) (196000) were purchased from Sigma‐ aldrich (Sigma, St. Louis, MO, USA). The MR‐1 gene knockdown plasmid (sc‐408848) and its homologous repair plasmid (HDR) were purchased from Santa Cruz Biotechnology (Dallas, TX, USA). Notch3 siRNAs (siG09820100759‐1‐5, siG09820100811‐1‐5) and its control siRNA were purchased from RiboBio (Guangzhou, China). Real‐time PCR master mix was purchased from Roche (Indianapolis, IN, USA). Peptide TMP22 was synthesized by Shenggong Biotechnology (Shanghai, China) and purified to a purity of 95% by high‐pressure liquid chromatography.

### Cell Culture

Human embryonic kidney cells HEK‐293T and human bronchial epithelial cells BEAS‐2B were preserved in the laboratory, while human lung cancer cell lines H1975, A549, H460, 95‐D, and H1299 were purchased from the Cell Culture Center of Peking Union Medical College (PUMC, Beijing, China). The human lung cancer cell PC‐9 was kindly presented by Dean Zhang Buchang of School of Life Sciences, Anhui University. H1975, A549, H460, and 95‐D cells were cultured in 1640 (MacGene, Beijing, China) containing 10% fetal bovine serum (Gibco). H1299 cells were cultured in 1640 containing 20% fetal bovine serum. HEK‐293T and PC‐9 cells were cultured in DMEM containing 10% fetal bovine serum.

All the cells were cultured in medium with 100 mg mL^−1^ streptomycin and 100 U mL^−1^ penicillin (MacGene) at 37 °C in 5% CO_2_. All cell lines were maintained and used at ≤20 passages.

### Immunohistochemistry (IHC)

First, paraffin sections were deparaffinized and heated in a pressure cooker for 10 min. After cooling to room temperature, sections were immersed for 5 min in PBS 3 times, and followed by endogenous peroxidase activity blocking with 3% H_2_O_2_. Then, non‐specific staining was blocked with 10% goat serum. And sections were incubated overnight with the first antibody (1:500‐1:200) at 4 °C and treated with the second antibody at 37 °C for 30 min. Sections were stained with DAB for 2 min and rinsed with deionized water to terminate the DAB reaction. Images were acquired with an optical microscope and analyzed with ImageJ software.

### Lung Cancer Tissue Microarray (TMA)

TMA containing 94 cases of lung cancer diseases was purchased from Alenabio (LUM961, Xi'an, China). It includes 4 cases of adenosquamous carcinoma, 1 case of adenocarcinoma, 2 cases of squamous carcinoma and the corresponding adjacent cancer tissues of the above 7 patients, 3 cases of adenosquamous carcinoma, 15 cases of adenocarcinoma, 21 cases of squamous carcinoma, 1 case of large cell carcinoma and the corresponding metastatic cancer tissues of the above 40 patients. In TMA, IHC was used to detect the expression of MR‐1 (HPA010134, 1:300; Sigma).

### Animal Models

The 6‐8‐week‐old BABL/c nude mice were purchased from Beijing Sibeifu Biotechnology Co., Ltd. To investigate the effects on lung colonization, the indicated A549 cells (1.2 × 10^7^) were intravenously injected, and metastases were observed after 14 days.

The transgenic mice are all C57BL/6N strain mice. *Sftpc‐Cre/ERT2* mice were purchased from Beijing Weishang Lide Biotechnology Co., Ltd. *Kras* ^LSL‐G12D^ and *Trp53* ^LSL‐R172H^ transgenic mice were purchased from Jiangsu Jicui Yaokang Biotechnology Co., Ltd. The *PNKD* gene knock‐in mice (*PNKD*‐KI) and *PNKD* gene knockout mice (*PNKD*‐KO) were constructed by Beijing Baiaosaitu Gene Biotechnology Co., Ltd.

When *Cre^ERT2^* transgenic mice also contain loxP‐flattened sequences, tamoxifen induced Cre mediated recombination leads to expression or deletion of sequences directed toward type II alveolar epithelial cells. The above mice were bred and their tails were taken at 2 weeks of age for genotype identification (Table S1, Supporting Information, for primer information), and the mice with the required genotype were selected. The targeted deletion or expression of genes was induced using tamoxifen (Sigma Aldrich, 06734, 75 mg kg^−1^) at 6 weeks of age. Computed Tomography (CT) and magnetic reso‐nance imaging (MRI) experiments were conducted by relevant technicians from the Institute of Zoology, Peking Union Medical College, Chinese Academy of Medical Sciences.

All animal procedures are conducted in accordance with the guidelines of the PUMC Biomedical Research Ethics Committee for Animal Care and Treatment.

### 5.1 Cultivation of Organoids

The mouse lung tumor tissue were cut into small pieces of approximately 5 mm, rinsed repeatedly with PBS buffer containing dual antibodies, and removed normal cells using EDTA chelation buffer at 4 °C. The treated tissue blocks were digested into individual cells in DMEM medium supplemented with type IV collagenase and type II dispersed enzyme at 37 °C for 45 min. After sieving with a 70 µm mesh, they were resuspended at 1000 rpm for counting.

After mixing individual cells with matrigel in a 24 well plate, it was solidified at 37 °C for 30 min. Then it was cultured in medium DMEM/F12 with HEPES, N2, B27, NAC, EGF, Wnt‐3α, Noggin, and R‐Spondin, cultured in an incubator containing 5% CO_2_ at 37 °C.

### Human Protein Atlas

Human Protein Atlas (HPA, https://www.proteinatlas.org) uses transcriptome and proteomics techniques to identify tumor type specific protein expression patterns differentially expressed in specific types of tumors^[^ ^73^ ^]^. In this study, we directly compared the differences in MR‐1 protein expression in lung cancer and adjacent tissues using IHC images via HPA.

### Survival Analysis

Using the GSE31210 dataset from the GEO database, patients were divided into high‐risk and low‐risk groups based on the median expression of *PNKD*. The risk curves for overall survival (OS) and release free survival (RFS) were plotted by GraphPad Prism 6.

### TCGA Database

After downloading the level 3 HTSeq‐FPKM (Fragments Per Kilobase per Million) format RNAseq data from the TCGA database (https://portal.gdc.cancer.gov/) for 594 lung adenocarcinoma patients and 420 lung squamous cell carcinoma patients (as of June 14, 2022), the *PNKD* [ENSG0000127838] was analyzed using R software (version 3.6.3). The pROC package is used to analyze and plot ROC curves, while the ggplot2 package is used to visualize differential expression (filtered/unfiltered paired samples). Significantly marked: *, *p* < 0.05; **, *p* < 0.01; ***, *p* < 0.001.

### Gene Set Enrichment Analysis (GSEA)

Enrichment analysis was conducted on the high and low expression populations of *PNKD* gene using the GSEA. In this study, the gene sets of GSEA v.4.0 c2.all.v7.4.symbols.gmt [Curated] and c2.cp.kegg. v7.4.symbols.gmt [Curated] was analyzed. *P*‐value<0.05 and the enrichment results of false detection rate (FDR) <0.25 were considered statistically significant. The visualization results delivered by Huada Gene (Shenzhen, China) were analyzed simultaneously.

### Ubibrowser

The E3 ubiquitin ligase was predicted using Notch3 as the substrate through the online website Ubibrowser (http://ubibrowser.bio‐it.cn/ubibrowser_v3/), and the results were visualizated through Cytoscape_ V3.9.1.

### Cluster X2.1 Sequence Alignment

According to the intracellular segments of Notch1‐3 (1757‐2555, 1699–2471, 1665–2321), Cluster X2.1 was used to do complete alignment. And the results were visualizated through the auxiliary software GeneDoc.

### Kaplan‐Meier Plotter

Using the Kaplan‐Meier plotter (http://kmplot.com/analysis/)^[^ ^74^ ^]^ and according to the expression of ITCH, patients were divided into high‐risk groups and low‐risk groups, the risk curves of Overall Survival (OS) were drawn.

### Protein Structure Prediction Analysis

Predictprotein (https://open.predictprotein.org/) and TMHMM (http://www.cbs.dtu.dk /services/TMHMM/) website can obtain many structural information related protein sequence, such as functional prediction, secondary structure, motif and domain^[^ ^75^, ^76^, ^77^ ^]^. We predicted the secondary structure of MR‐1 protein through the above website. The tertiary structure of MR‐1, NICD3 and TMP22 was predicted through I‐TASSER server and AlphaFold v2.3.2.^[^ ^78^, ^79^, ^80^, ^81^ ^]^

### Western Blot

The cells were lysed with RIPA buffer (containing 1% PMSF). Subsequently, BCA method was used to determine the protein concentration. Proteins were isolated using SDS‐polyacrylamide gel electrophoresis (SDS‐PAGE) and transferred to PVDF membrane (Millipore, Burlington, Massachusetts, USA). Then use 5% skim milk powder to block. The membranes were immunoblotted with the indicated antibody, and the enhanced HRP substrate chemiluminescence solution (ECL) was used for imaging in the FluorChem HD2 imaging system (Protein Simple, CA, USA).

### Transwell

After the chambers were balanced with serum‐free medium for 30 min, the cells in logarithmic growth phase after transient transfection for 24 h were digested. The upper compartment was inoculated with serum‐free medium, and the lower compartment was inoculated with 20% FBS. According to the needs of the experiment, 4% paraformaldehyde was used for fixation at 24 h and 48 h respectively, and crystal violet staining was used and the upper chamber with cotton swabs to remove cells inside. After the completion, the chamber was observed and photographed with Olympus inverted fluorescence microscope. The chamber was cut off with a blade, dissolved in 33% acetic acid, and the absorbance value at 570 nm was measured with a microplate analyzer. The migration experiment was conducted by product (3422) purchased from Corning (Steuben County, New York, USA). For H1299 and H460 cells, the number of cells inoculated in the upper chamber is 10 000 and 25 000, respectively. The migration experiment was conducted by product (354480) purchased from Corning (Steuben County, New York, USA). For H1299 and H460 cells, the number of cells inoculated in the upper chamber is 15 000 and 150 000, respectively.

### Cytoplasmic Nucleus Separation Experiment

The Cytoplasmic Nucleus Separation Kit (#P1201, Pulilai Gene Technology, Beijing, China) was used to isolate cytoplasmic nuclear proteins. Western blot was used to detect the expression of related proteins.

### Wound‐Healing Experiment

For ≈90% of the cells in the treated 6‐well plate, the cells were vertically scratched with a 1 mL pipette tip, discarded the culture medium, washed with PBS 2–3 times, added 3% FBS culture medium, and cultured in an incubator containing 5% CO_2_ at 37 °C. Take samples at 0, 24, 48, 72, 96 h and other time points, observe under the microscope and take photos for preservation. The scratch healing rate and cell migration ability were analyzed ImageJ software.

### CCK8 Assay Experiment

A 10% CCK‐8 solution was added and incubated at 37 °C for 2 h. The optical density at 450 nm was measured using a spectrophotometer.

### Adhesion Experiment

After coating 10 µg mL^−1^ FN at 37 °C in a 96 well plate for 45 min, the logarithmic growth phase cells were digested and resuspended in the corresponding culture medium without serum with a final density of 10^5^ per 100 µL in 96 well plates coated with FN. Incubate 6 h as a control. After reaching the corresponding time point, remove the liquid from the corresponding well, use PBS suction and blow once. Finally, the CCK8 method was used to detect adhesive cells, and calculate the adhesion rate.

### Colony Formation Assay

Cells were seeded in six‐well plates at a density of 500–1000 cells per well. After culture with fresh medium for 1–2 weeks, the cells were fixed in anhydrous methanol for 10 min and stained with 0.1% crystal violet for 10 min. Colonies containing more than 50 cells were counted and scanned.

### Quantitative Real‐Time Polymerase Chain Reaction (qRT‐PCR)

Total RNA was extracted from cells using TRIzol reagent (Invitrogen). qRT‐PCR was performed using SYBR Green Master Mix (Roche, Basel, Switzerland). All samples were quantified using the ABI 7500Fast real‐time PCR detection system (Applied Biosystems, Foster City, CA, USA). Data were analyzed by the 2^−ΔΔCT^ method and GAPDH was used as a housekeeping gene.

The following primers were used:

HES1 forward:5 ‘ – ATGGAGAAAAATTCCTCGTCCC ‐3 ’
HES1 reverse:5 ‘ – TTCAGAGCATCCAAAATCAGTGT ‐3 ’
HEY1 forward:5 ‘ ‐GAAACTTGAGTTCGGCTCTAGG‐3 ’
HEY1 reverse:5 ‘ ‐GCTTAGCAGATCCTTGCTCCAT‐3 ’
HEY2 forward:5 ‘ ‐AGGGGGTAAAGGCTACTTTGA‐3 ’
HEY2 reverse:5 ‘ ‐TGGCGCAAGTGCTGAGATG‐3 ’
GAPDH forward:5 ‘ ‐GGAGCGAGATCCCTCCAAAAT‐3 ’
GAPDH reverse:5 ‘ ‐GGCTGTTGTCATACTTCTCATGG‐3 ’

### Co‐Immunoprecipitation, Co‐IP

To 1 mL of the BCA protein assay kit (Beyotime, Shanghai, China) quantified cell lysate (9803s, CST, containing 1% PMSF), 2 µL of primary antibody was added and incubated for 2 h at 4 °C. After that, 20 µL of Protein A/G PLUS‐Agarose (Santa Cruz, TX, USA sc‐2003) was added. The tubes were capped and incubated at 4 °C on a rocking platform overnight. After incubation, the tubes were centrifuged at 3000 rpm for 3 min, discarded the supernatant, washed three times with Co‐IP washing solution, and added 20 µL 2x Loading Buffer, denatured for 10 min. The proteins were separated using SDS‐PAGE.

### SPR Experiment

Reconstruct the NICD3 sequence onto the pET28a vector, transform it into BL21 responsive cells, and shake the bacteria. After induction by IPTG, the protein was purified at ÄKTA pure 25 L (Massachusetts, USA). Fix the purified NICD3 onto the surface of the Ni‐NTA chip (Reichert lnc., USA). Dissolve TMP22 in PBST (Containing 0.5% Tween) buffer, with a maximum concentration of 100 mM, and double dilute for 6 concentrations. Set the flow rate to 25 µL min^−1^, contact and dissociation times were set to 100 and 300 seconds, respectively. The Reichert4SPR system (Buffalo, New York, USA) was used to determine the dissociation rate constant (Kd), association rate constant (Ka) and equilibrium dissociation constant (KD).

## Conflict of Interest

The authors declare no conflict of interest.

## Author Contributions

W.Z., R.S. and W.Z. conceptualized the study. W.Z., R.S., and Y.Y. participated in the design, supervision, and coordination of the study. Y.Y. directed the design of peptide TMP22 and performed some of the experiments. WX Zhao was the major contributors in writing the manuscript and doing experiments. Y.Y., YL., H.C., M.W., Z.Z., M.C., C.Z., X.X., and X.Z. investigated and helped analyze the data. R.S. and Y.Y. reviewed the manuscript. R.S., Y.Y., and W.Z. obtained the funding.

## Supporting information

Supporting Information

## Data Availability

The data that support the findings of this study are available from the corresponding author upon reasonable request.
