# Supplementary material for: Myofibrillogenesis Regulator‐1 Regulates the Ubiquitin Lysosomal Pathway of Notch3 Intracellular Domain Through E3 Ubiquitin‐Protein Ligase Itchy Homolog in the Metastasis of Non‐Small Cell Lung Cancer
Source: Adv Sci (Weinh). 2024 Feb 11;11(15):2306472. doi: 10.1002/advs.202306472 (PMC11022719; doi:10.1002/advs.202306472)
Supplement: Supplementary file 1 — Supporting Information [file ADVS-11-2306472-s001.pdf]

ADVANCED SCIENCE
Open Access

Supporting Information

for *Adv. Sci.*, DOI 10.1002/advs.202306472

Myofibrillogenesis Regulator-1 Regulates the Ubiquitin Lysosomal Pathway of Notch3 Intracellular Domain Through E3 Ubiquitin-Protein Ligase Itchy Homolog in the Metastasis of Non-Small Cell Lung Cancer

Wenxia Zhao, Yang Li, Hanzeng Cheng, Mengyan Wang, Zhishuo Zhang, Meilian Cai, Cong Zhao, Xiaoming Xi, Xiaojun Zhao, Wuli Zhao, Yajun Yang* and Rongguang Shao**

Supporting Information

Myofibrillogenesis Regulator-1 Regulates the Ubiquitin Lysosomal Pathway of Notch3-ICD through ITCH in the Metastasis of Non-Small Cell Lung Cancer

Wenxia Zhao[a], Yang Li[a], Hanzeng Cheng[b], Mengyan Wang[a,c], Zhishuo Zhang[d,e], Meilian Cai[a], Wuli Zhao[a,], Yajun Yang[b,]*, Rongguang Shao[a,]**

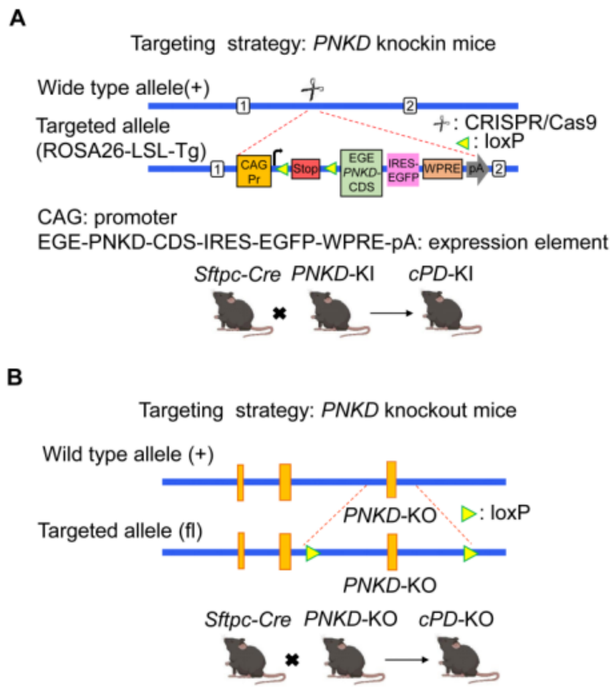

Figure S1 Construction of transgenic mice. (**A**) A schematic of the strategy for generating *PNKD* knock-in mice (*PD*-KI). (**B**) A schematic of the strategy for generating *PNKD* knockout mice (*PD*-KO).

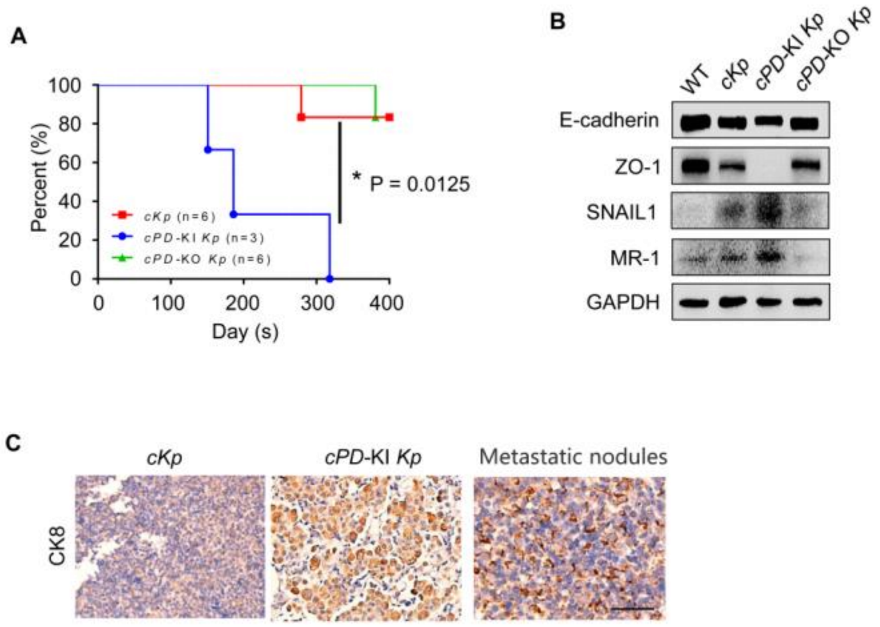

Figure S2 MR-1 promotes malignant progression of NSCLC in transgenic mice. **(A)** OS curve analysis for mice with the indicated genotypes at the indicated ages. Log-rank (Mantel-Cox) test. *$p < 0.05$. (**B)** Expression of epithelial mesenchymal transition (EMT) related proteins in lung tissue of transgenic mice with the indicated genotypes. (**C)** Immunohistochemical (IHC) of CK8 in lung tumor tissue and metastatic thyroid tissue of transgenic mice with the indicated genotypes. Scale bar, 400 μm.

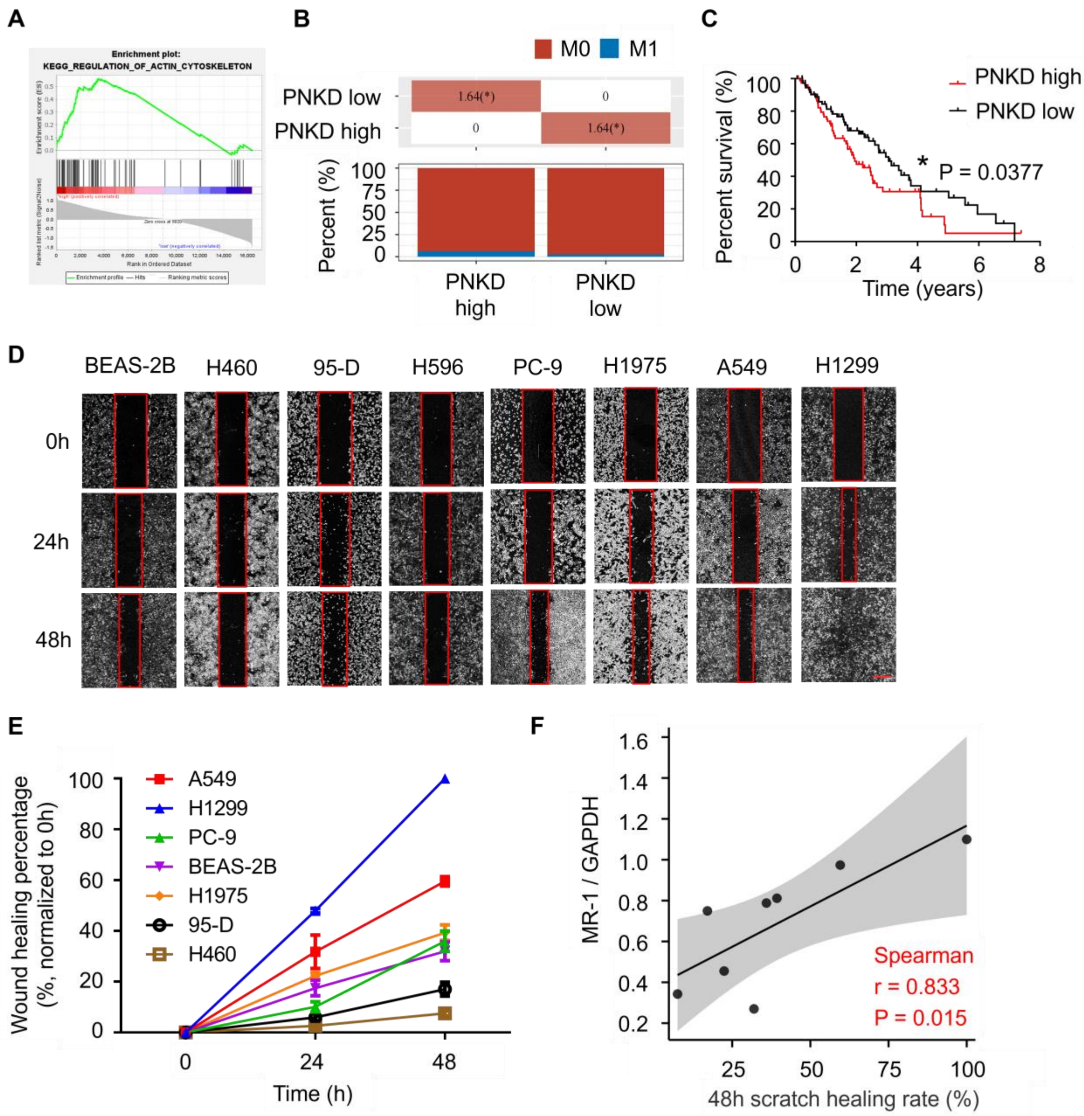

Figure S3 The expression of MR-1 is positively correlated with lung cancer metastasis. (**A**) A GSEA analysis of gene expression profiles of NSCLC patients with high or low expression of *PNKD* in the TCGA lung cancer datasets. (**B**) An analysis of the correlation between *PNKD* and distant metastasis through TCGA lung cancer datasets. **p* < 0.05. (**C**) Kaplan Meier analysis of the survival rate of 153 NSCLC patients with lymph node metastasis, who were divided into low or high subgroups based on median *PNKD* expression. **p* < 0.05. (**D, E**) Representative images of wound healing experiments for the normal bronchial epithelial cell line (BEAS-2B) and seven NSCLC cell lines (H460, 95-D, H596, PC-9, H1975, A549, H1299). Scale bar, 500 µm. The data was shown as mean ± SEM (n = 3). (**F**) Spearman correlation analysis between the relative expression level of MR-1 and the 48 h scratch healing rate of eight cell lines.

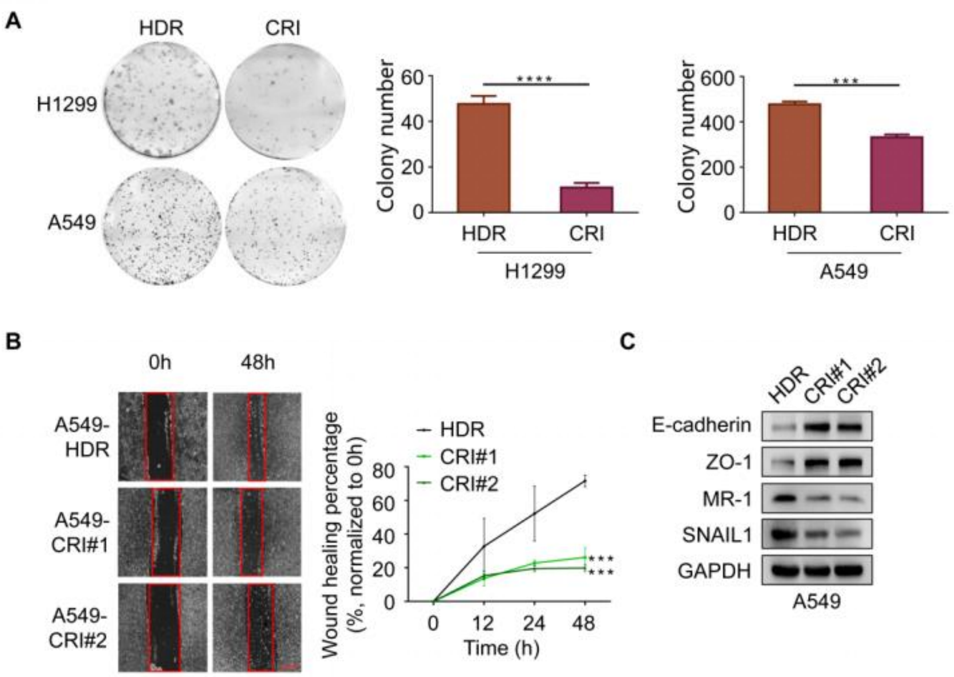

Figure S4 (A) Colony formation assays assessed the colony formation capacity of stable MR-1-knockdown H1299 and A549 cells. Colony formation rate = (numbers of colonies / numbers of seeded cells) × 100%. The colony formation relative to control group was detected. The data was shown as mean ± SEM (n = 3). Unpaired t-test. ***$p < 0.005$; ****$p < 0.001$. (**B**) Representative images of wound-healing experiments within 48 hours were performed to detect cell migration in stable MR-1-knockdown A549 cells. ***$p < 0.005$; ****$p < 0.001$. (**C**) Western blot analysis of EMT related proteins expression in stable MR-1-knockdown A549 cells.

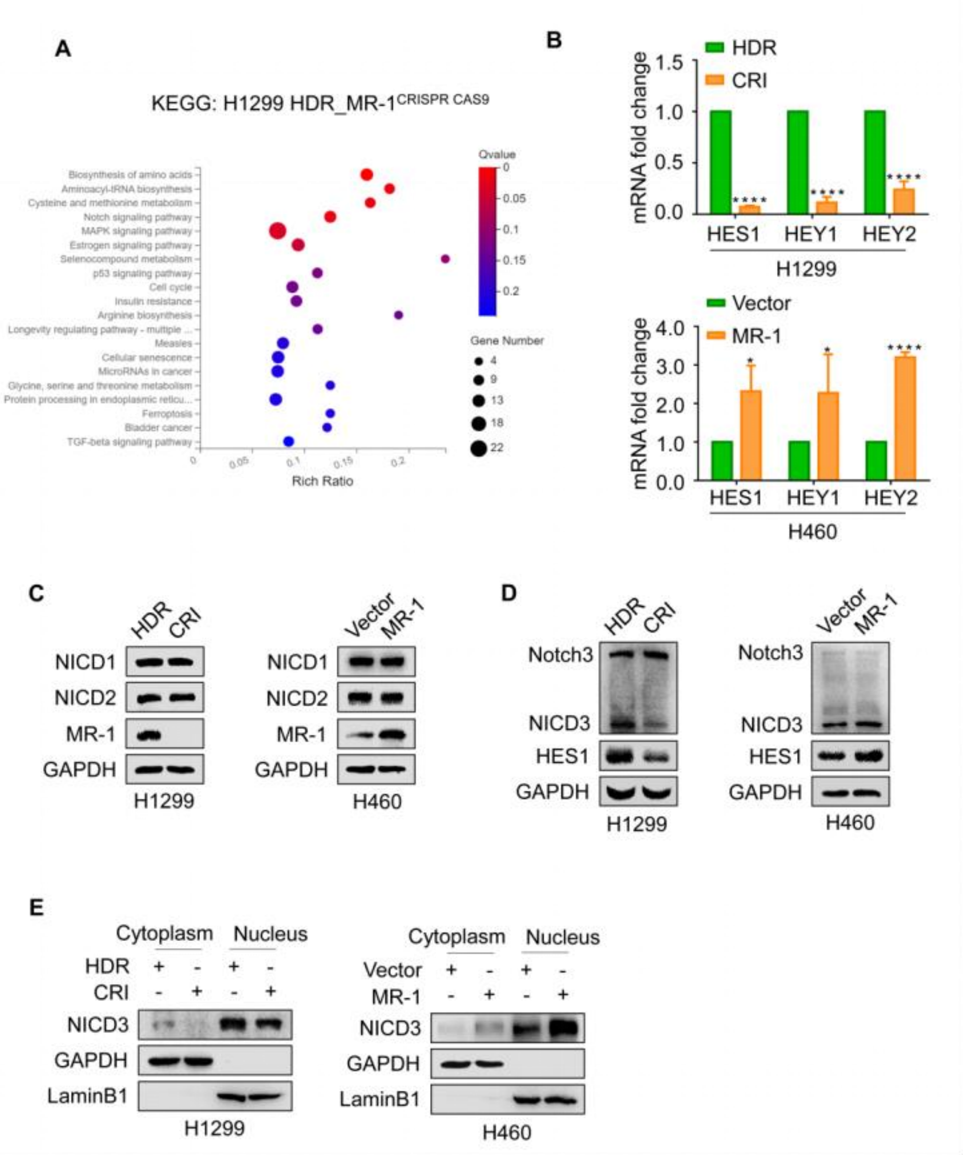

Figure S5 MR-1 affects the expression and nuclear localization of NICD3. **(A)**Enrichment Analysis of KEGG Pathway in stable MR-1-knockdown H1299 cells. (**B)** The mRNA levels of Notch signaling pathway target genes in stable MR-1-knockdown or MR-1-overexpression H1299 and H460 cells. The data are shown as mean ± SEM (n = 3). Two-way ANOVA. *p < 0.05; ****p < 0.001. (**C, D)** Western blot analysis of NICD1-3 and target gene HES1 expression in stable MR-1-knockdown or MR-1-overexpression H1299 and H460 cells. (**E)** Cytoplasmic Nucleus Separation Experiment were performed to detect Nuclear Localization of NICD3 in stable MR-1-knockdown or MR-1-overexpression H1299 and H460 cells.

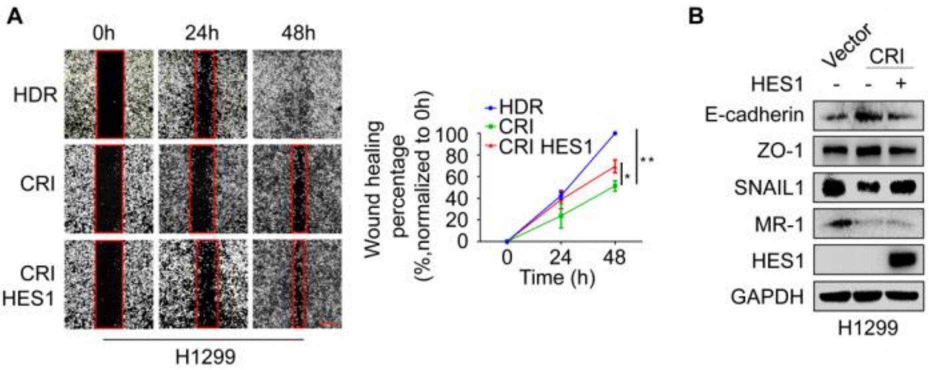

Figure S6 (A, B) Overexpressing of HES1 reversed cell scratch healing ability (**A** Scale bar, 500 μm.) and EMT related proteins expression (**B**) in stable MR-1-knockdown H1299. The data are shown as mean ± SEM (n = 3). Two-way ANOVA. *$p < 0.05$; **$p < 0.01$.

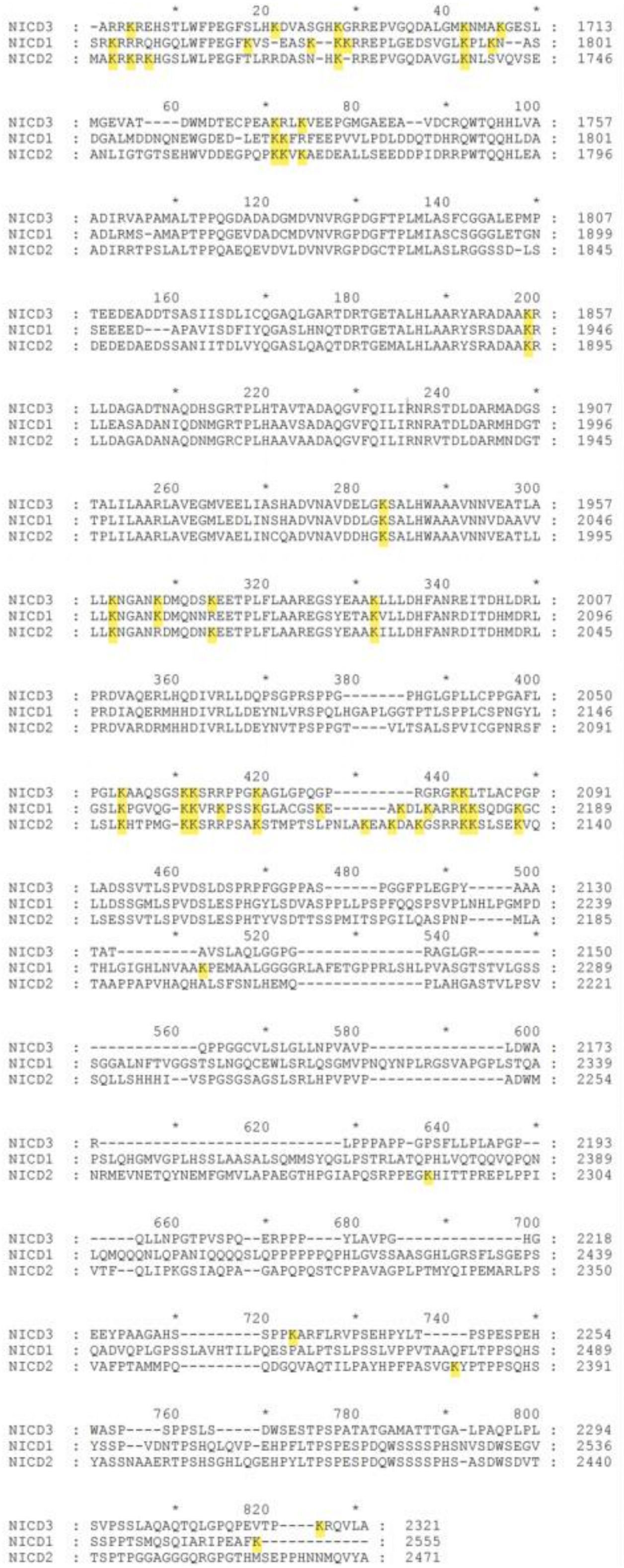

Figure S7 Clustal X2.1 alignment of NICD sequences. Notch amino acid sequences were compiled from the NCBI GenBank database. After visualization by Genedoc, significant sequence alignment is shown for human NICD1-3. Lysine residues (K), which are potential targets for ubiquitin ligation, are highlighted to show their relative conservation among the three NICDs. At the right, amino acid residue counts reflect their positions throughout the protein sequence.

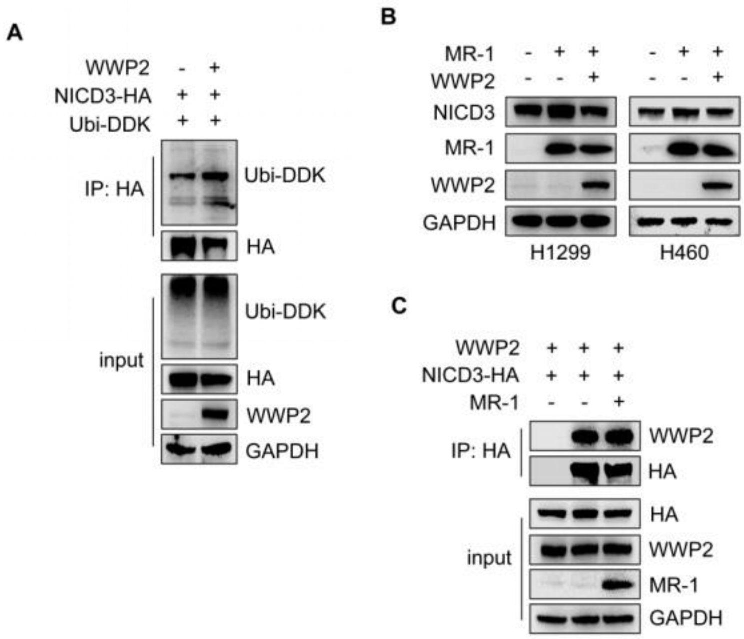

Figure S8 (A) pCDNA3.1-HA-NICD3, pCMV3-WWP2 and pCMV3-DDK ubiquitin plasmids were co transfected into HEK-293T cells for 48 h, and the cell extracts were IP with anti-HA Ab. Ubiquitinated NICD3 was detected by Western blot. (**B)** After transient transfection of pCDNA3.1-MR-1, pCMV3-WWP2 plasmids in H1299 and H460 cells for 48 h, the effects of MR-1 and WWP2 on NICD3 expression were detected by Western blot. (**C)** HEK-293T cells were transfected with the indicated plasmids for 48 h. Cell extracts were IP with an anti-HA Ab. The interaction between WWP2 and NICD3 was detected by Western blot.

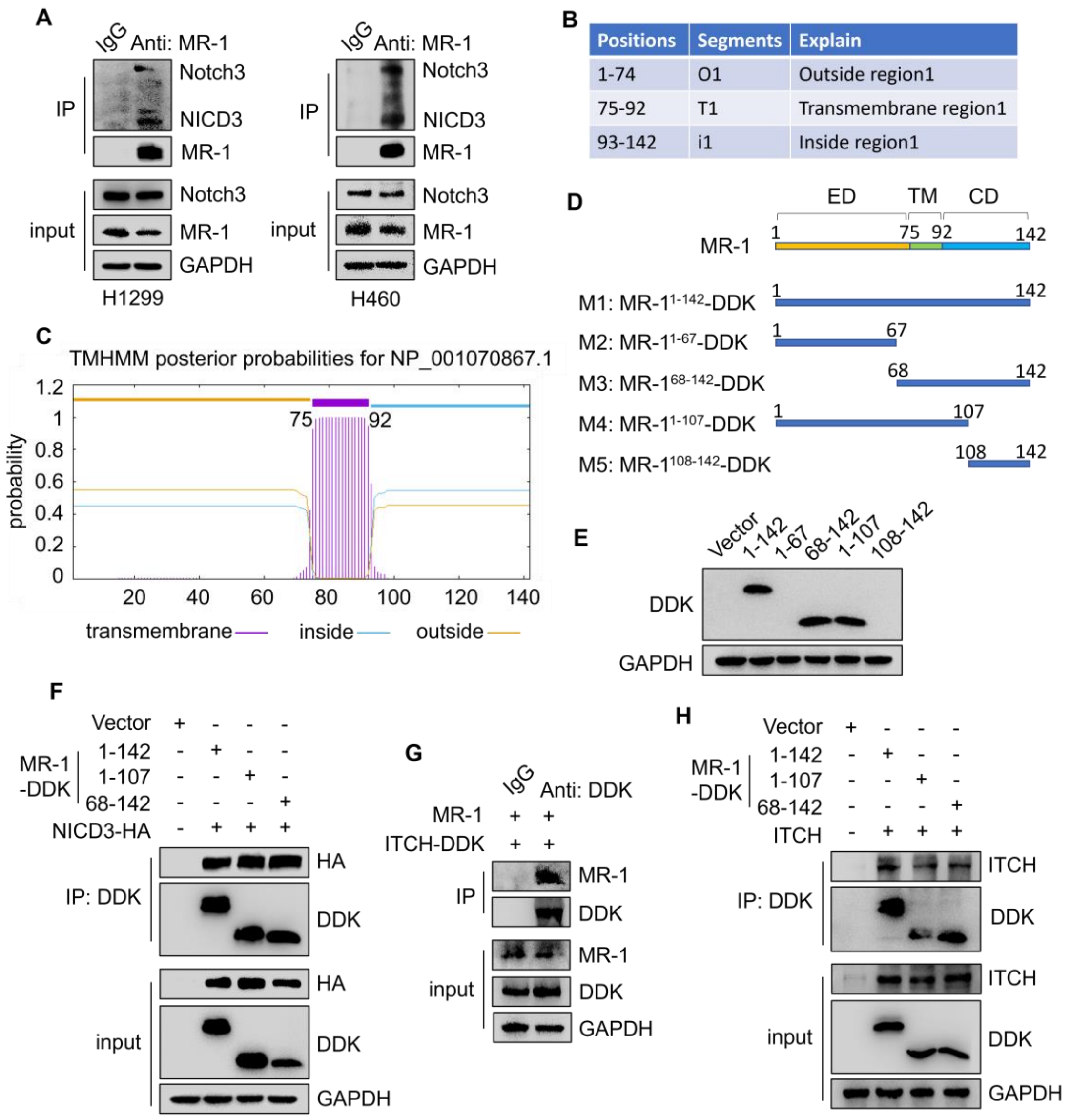

Figure S9 The binding of MR-1 and NICD3. **(A)** Endogenous binding of Notch3/NICD3 and MR-1 was detected in H1299 and H460 cells by Western blot. **(B)** Predictprotein platform predicted the transmembrane domain of MR-1. **(C)** Biology online software tool TMHMM Server, v.2.0 predicted the transmembrane domain of MR-1. **(D)** Schematic diagram of MR-1 region. **(E)** Detecting the availability of truncated plasmids by Western blot. **(F)** After transient transfection of pCMV6-DDK-MR-1 truncated plasmids and pCDNA3.1-HA-NICD3 plasmid in HEK-293T cells for 48 h, the interaction of NICD3 and MR-1 region was detected by Western blot. **(G)** After transient transfection of pCMV6-DDK-MR-1 plasmids and pCMV6-ITCH plasmid in HEK-293T cells for 48 h, exogenous binding of NICD3 and MR-1 was detected in HEK-293T cells by Western blot. **(H)** After transient transfection of pCMV6-DDK-MR-1 truncated plasmids and pCMV6-ITCH plasmid in HEK-293T cells for 48 h, the interaction of NICD3 and MR-1 region was detected by Western blot.

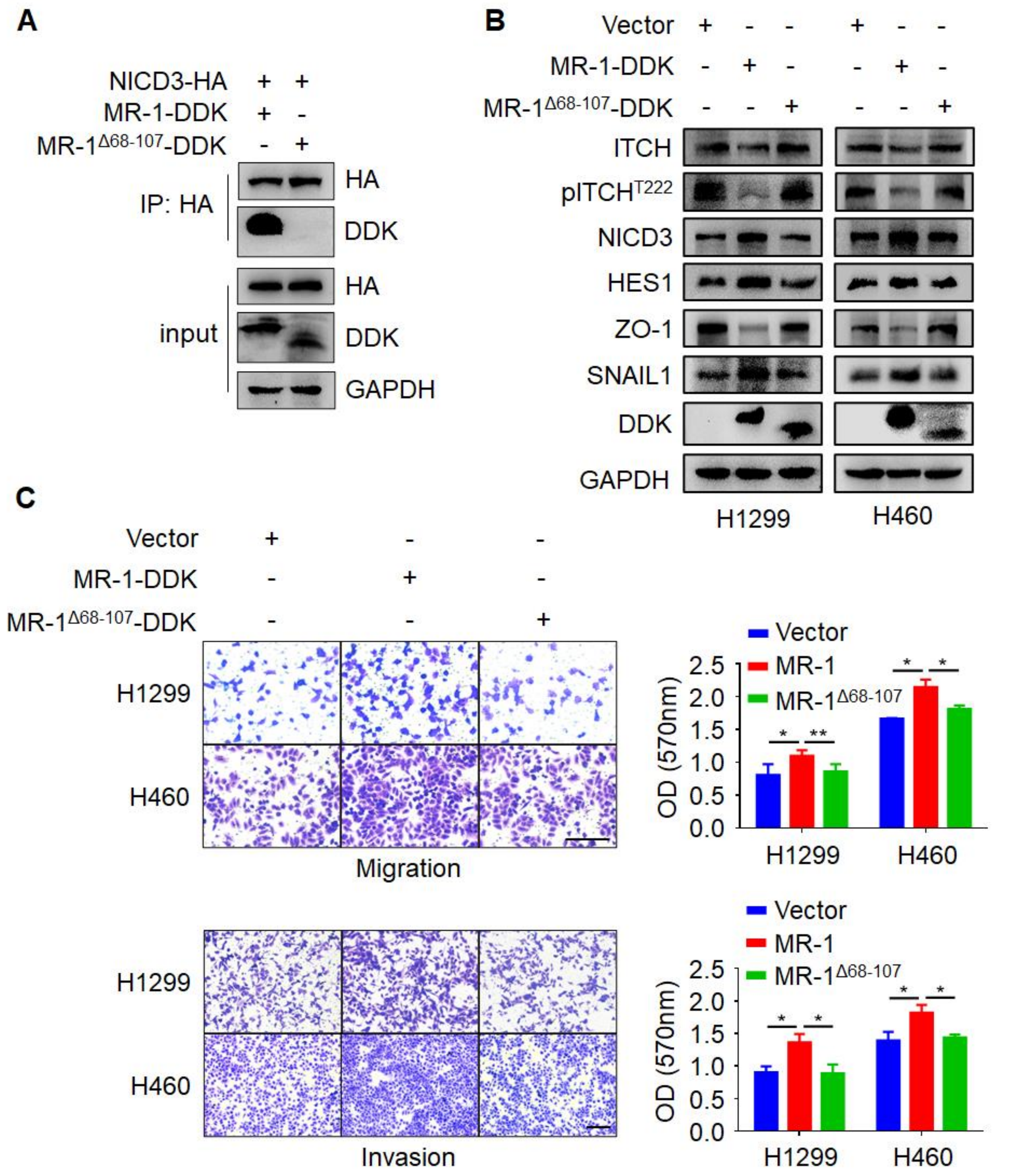

Figure S10 (A) After transient transfection of pCMV6-DDK-MR-1 or 68-107 fragment-deficient MR-1 plasmid and pCDNA3.1-HA-NICD3 plasmid in HEK-293T cells for 48 h, the interaction of NICD3 and MR-1 or 68-107 fragment-deficient MR-1 was detected by Western blot. **(B)** Overexpressing MR-1 or 68-107 fragment deletion by transient transfection detected NICD3/ITCH and EMT related proteins expression, cell migration ability (**C** Scale bar, 200 μm.) and cell invasion ability (**C** Scale bar, 600 μm.). The data was shown as mean ± SEM (n = 3). Two-way ANOVA. *p < 0.05; **p < 0.01.

A

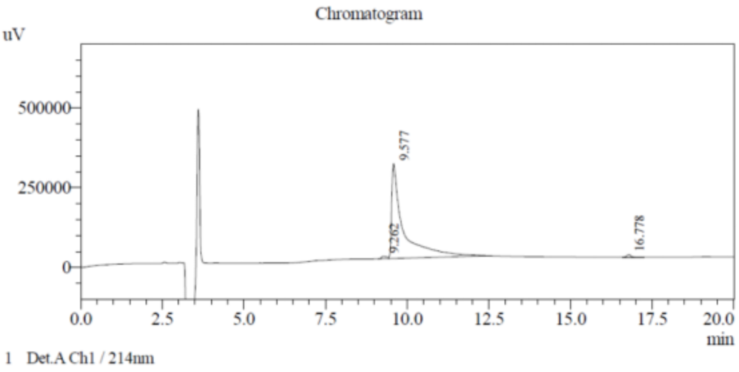

Peak	Retention time	Peak area	Peak height	Peak area %
1	9.262	86091	7551	1.139
2	9.577	7415563	297166	98.080
3	16.778	59092	7553	0.782
In total		7560745	312271	100.000

B

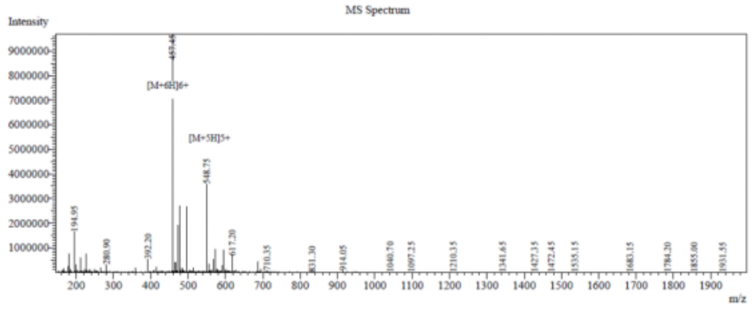

C

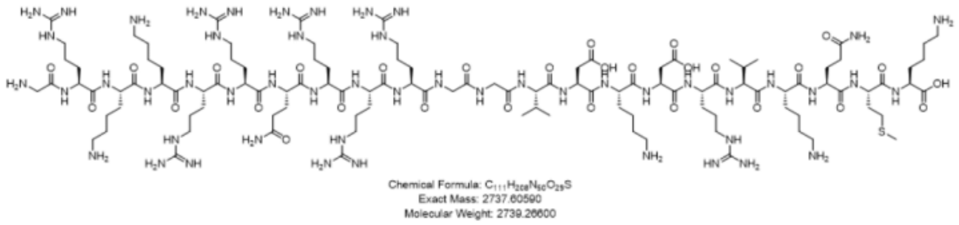

Figure S11 (A) Purification of peptide with HPLC. UV detection at 214 nm; gradient elution: 12% Phase B to 32% Phase B (Phase A= 0.1% TFA in water, Phase B= 0.1% TFA in acetonitrile), Flow rate = 1.0 mL/min. Purity: 98.08%. (**B)** Identification of peptide with mass spectrometry. (**C)** Structure of peptide. (H-Gly-Arg-Lys-Lys-Arg-Arg-Gln-Arg-Arg-Arg-Gly-Gly-Val-Asp-Lys-Asp-Arg-Val-Lys-Gln-Met-Lys-OH)

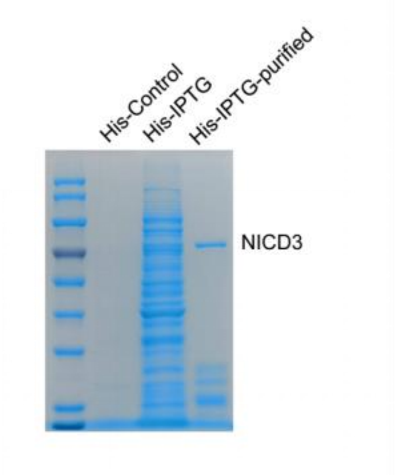

Figure S12 NICD3 protein was induced by IPTG and purified, which was stained with Coomassie Brilliant Blue.

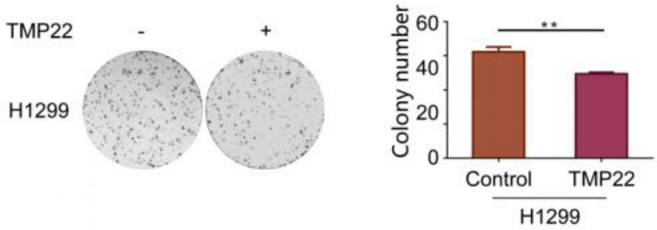

Figure S13 Colony formation assays assessed evaluated the impact of TMP22 on the colony formation ability of H1299. Colony formation rate = (numbers of colonies / numbers of seeded cells) × 100%. The colony formation relative to control group was detected. The data was shown as mean ± SEM (n = 3). Unpaired t-test. $^{**}p < 0.01$.

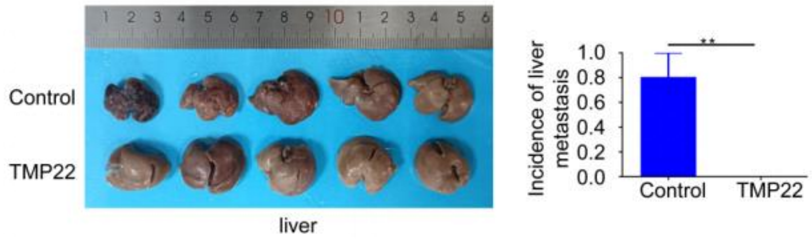

Figure S14 A549 cells (1×10^7 cells) were injected intravenously into nude mice (n = 5 per group), followed by daily intravenous injection of normal saline five days later. The liver tissues after metastasis were shown.

Table. S1 Primer information of transgenic mice.

Identifying genotypes	Primer	Primer sequence	Product size
Kras$^{LSL\text{-}G12D}$, *Trp53*$^{LSL\text{-}R172H}$	Trp53-KI-5tF1	GGTTGGCCGAACTCAATCCC	KI: 904 bp
	Trp53-KI-5tR1	GTCACACCACAGAAGTAAGGTTCC	
	Trp53-WT-tF1	CCTCCCTGATTACCTGTTCCTTG	WT: 618 bp
	Trp53-WT-tR1	TGTCTCCTGGCTCAGAGGGAG	
	Kras-G12D-geno-5F1	TAACCTCCTAATCCTGTTCCCTACCA	KI: 1229 bp
	Kras-G12D-geno-5R3	TGCATCCATCTAGATCTCGATCG	
	Kras-G12D-geno-wtF1	ACACCAGCTTCGGCTTCCTA	WT: 425 bp
	Kras-G12D-geno-wtR1	ATTCCGAATTCAGTGACTACAGATG	
sftpc-Cre	Cre-ERT2-F	CGCTAAGGATGACTCTGG	Mut: 334 bp
	Cre-ERT2-R	CAACAAGGCACTGACCAT	
PNKD-KI	PNKD-KI-F	AGTCGCTCTGAGTTGTTATCAG	Mut: 469 bp
	PNKD-KI-R	GTCAATGGAAAGTCCCTATTGGCGT	
PNKD-KO	EGE-LS-095-5'loxP-F	TTGCGTTGTGTAGAAGCTAGACGTG	WT: 290
	EGE-LS-095-5'loxP-R	CAACTGAAAGAGGGCATTGGTAAGG	Mut: 428
